# ACTL6A: unraveling its prognostic impact and paving the way for targeted therapeutics in carcinogenesis

**DOI:** 10.3389/fmolb.2024.1387919

**Published:** 2024-05-30

**Authors:** Refaat A. Eid, Farag Mamdouh, Waleed K. Abdulsahib, Dalal Sulaiman Alshaya, Fawziah A. Al-Salmi, Maha Ali Alghamdi, Ibrahim Jafri, Eman Fayad, Ghadi Alsharif, Mohamed Samir A. Zaki, Mohammed A. Alshehri, Ahmed E. Noreldin, Muhammad Alaa Eldeen

**Affiliations:** ^1^ Pathology Department, College of Medicine, King Khalid University, Abha, Saudi Arabia; ^2^ Biotechnology Division, Zoology Department, Faculty of Science, Benha University, Banha, Egypt; ^3^ Pharmacology and Toxicology Department, College of Pharmacy, Al Farahidi University, Baghdad, Iraq; ^4^ Department of Biology, College of Science, Princess Nourah Bint Abdulrahman University, Riyadh, Saudi Arabia; ^5^ Biology Department, College of Sciences, Taif University, Taif, Saudi Arabia; ^6^ Department of Biotechnology, College of Sciences, Taif University, Taif, Saudi Arabia; ^7^ Department of Clinical Laboratory Sciences, College of Applied Medical Sciences, King Saud Bin Abdulaziz University for Health Sciences, Jeddah, Saudi Arabia; ^8^ Department of Biomedical Research, King Abdullah International Medical Research Center, Jeddah, Saudi Arabia; ^9^ Anatomy Department, College of Medicine, King Khalid University, Abha, Saudi Arabia; ^10^ Department of Child Health, College of Medicine, King Khalid University, Abha, Saudi Arabia; ^11^ Department of Histology and Cytology, Faculty of Veterinary Medicine, Damanhour University, Damanhour, Egypt; ^12^ Cell Biology, Histology and Genetics Division, Zoology Department, Faculty of Science, Zagazig University, Zagazig, Egypt

**Keywords:** ACTL6A, oncogene, carcinogenesis, prognosis, pan-cancer

## Abstract

**Introduction:** Increased Actin-like 6A (ACTL6A) expression is associated with various cancers, but its comprehensive investigation across different malignancies is lacking. We aimed to analyze ACTL6A as a potential oncogene and therapeutic target using bioinformatics tools.

**Methods:** We comprehensively analyzed ACTL6A expression profiles across human malignancies, focusing on correlations with tumor grade, stage, metastasis, and patient survival. Genetic alterations were examined, and the epigenetic landscape of ACTL6A was assessed using rigorous methods. The impact of ACTL6A on immune cell infiltration in the tumor microenvironment was evaluated, along with molecular docking studies and machine learning models.

**Results:** Our analysis revealed elevated ACTL6A expression in various tumors, correlating with poor prognostic indicators such as tumor grade, stage, metastasis, and patient survival. Genetic mutations and epigenetic modifications were identified, along with associations with immune cell infiltration and key cellular pathways. Machine learning models demonstrated ACTL6A's potential for cancer detection.

**Discussion:** ACTL6A emerges as a promising diagnostic and therapeutic target in cancer, with implications for prognosis and therapy. Our study provides comprehensive insights into its carcinogenic actions, highlighting its potential as both a prognostic indicator and a target for anti-cancer therapy. This integrative approach enhances our understanding of ACTL6A's role in cancer pathogenesis and treatment.

## Graphical Abstract

**Figure:**
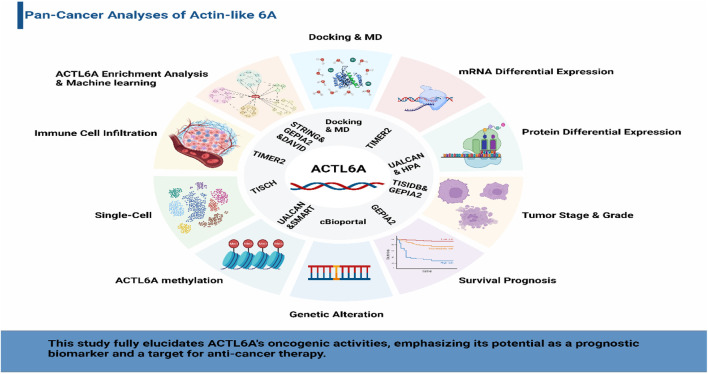
Decoding ACTL6A: a graphical abstract of its oncogenic significance, prognostic impact, and therapeutic potential in diverse human cancers through comprehensive bioinformatics analysis.

## 1 Introduction

Cancer, a pervasive global health challenge, ranks as the second leading cause of mortality worldwide, claiming the lives of over 8 million people annually, with projections indicating a staggering 50% increase in incidence in the coming decades (Bray et al., 2013). Characterized by unbridled cellular proliferation, invasion, and evasion of immune surveillance, the intricate understanding of cancer’s molecular underpinnings is imperative for developing effective therapeutic interventions (Hanahan and Weinberg, 2011). The landscape of cancer research has been revolutionized by continuous advancements in genomics, catalyzed by the introduction of a high-quality human reference genome two decades ago (Wheeler and Wang, 2013). The subsequent evolution of sequencing technologies and informatics tools has solidified genomics as a cornerstone in cancer research (ICGC/TCGA Pan-Cancer Analysis of Whole Genomes Consortium, 2020; Cortés-Ciriano et al., 2020). Notable databases and bioinformatics tools such as the Tumor Immune Estimation Resource, version 2 (TIMER2.0), GEPIA2, TNMplot, TISIDB, UALCAN, Human Protein Atlas (HPA), Kaplan-Meier (KM) plotter, cBioPortal, SMART, Tumor Immune Single-Cell Hub (TISCH), STRING, and Database for Annotation, Visualization, and Integrated Discovery (DAVID), along with docking and molecular dynamics (MD) tools like AutoDockTools and GROMACS-2023.1, have significantly enhanced the determination of crucial tumor indications and potential treatment targets (Ramos et al., 2020; Park et al., 2022; Hassan et al., 2023).

Simultaneously, the advent of immunotherapy, particularly immune checkpoint inhibitors (ICIs) like α PD-1 and α CTLA-4, has ushered in a new era in cancer treatment, prompting a surge in investigations into the intricate interplay between cancer-regulating genes and the immune system (Wu et al., 2019). The intersection of genomics and immunotherapy has become a focal point in contemporary cancer research, aiming to unravel the complexities of tumor-immune interactions.

**FIGURE 1:**
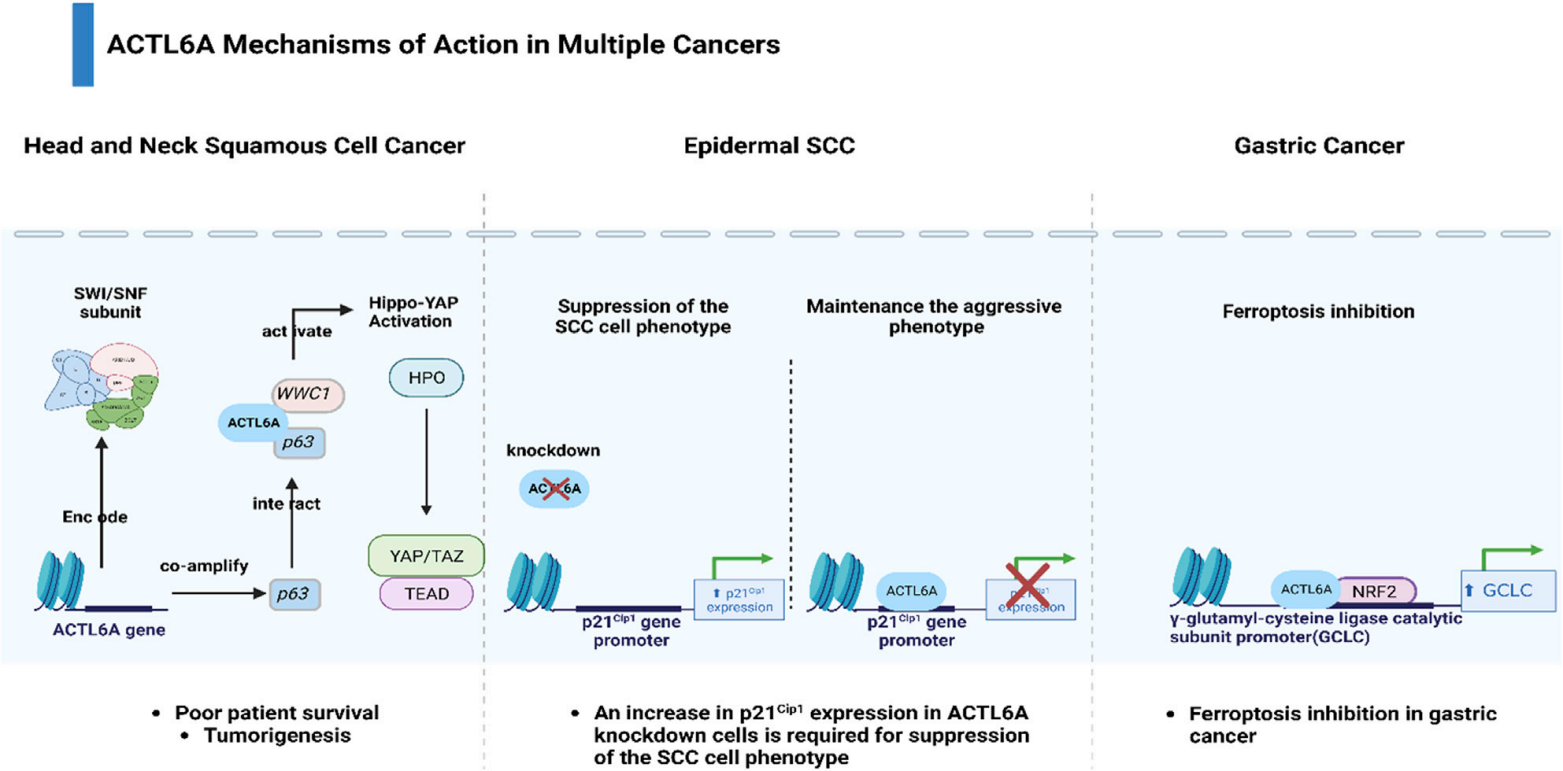
Diverse mechanisms of action of ACTL6A across multiple cancers.

Within the genomic landscape of cancer, mutations in genes encoding chromatin regulatory proteins, particularly subunits of the SWI/SNF chromatin-remodeling complexes, have emerged as prominent features, collectively accounting for nearly 25% of all cancers (Kadoch et al., 2013; Shain and Pollack, 2013). Despite their designation as tumor suppressors, these complexes are frequently mutated or lost in tumors, creating a permissive environment for cancer development (Yang Y.-L. et al., 2020; Chen et al., 2020). Among the key players in this context is Actin-like protein 6A (ACTL6A), which not only interacts with the SWI/SNF complex to activate the Brg1 ATPase but also acts independently to impact cancer cell survival, stem cell regulation, and metastasis (Krasteva et al., 2012; Taulli et al., 2014; Lu et al., 2015; Zhu et al., 2017). Moreover, ACTL6A has been reported to be related to the tumorigenesis of several cancers (Meng et al., 2017). Zeng et al. reported that ACTL6A exhibited protumor function and activated the epithelial-to-mesenchymal transition (EMT) in colon cancer (Zeng et al., 2018). In addition, Shuai Xiao et al. showed that ACTL6A promoted metastasis and EMT by activating SOX2/Notch1 signaling in hepatocellular carcinoma (Xiao et al., 2016). Saladi et al. demonstrated that ACTL6A was commonly amplified and highly expressed along with TP63, and together, they regulated WWC1 to facilitate oncogenic YAP activity. This coordination has implications for the prognosis of patients with head and neck squamous cell carcinoma (Saladi et al., 2017). ACTL6A was found to inhibit the activity of the p21Cip1 promoter, resulting in decreased production of the p21Cip1 protein. This mechanism helps to sustain the aggressive characteristics of epidermal squamous cell carcinoma (Shrestha et al., 2020). Fang et al. found that ACTL6A protects gastric cancer cells against ferroptosis through induction of glutathione synthesis (Yang et al., 2023) (Figure 1).

Despite its diverse implications in various cancers, a comprehensive investigation into the multifaceted roles of ACTL6A is conspicuously absent. This study aims to bridge this gap by conducting an exhaustive examination of ACTL6A expression patterns across diverse human tumors. Beyond expression profiling, we delve into the activation status of ACTL6A, its influence on immune cell infiltration, genetic modifications, methylation patterns, and its prognostic significance within the intricate milieu of the tumor microenvironment (TME). This holistic approach is designed to provide a nuanced and comprehensive understanding of ACTL6A’s dynamic involvement in tumor progression. Furthermore, the study seeks to explore the therapeutic potential of targeting ACTL6A, offering novel avenues for developing antitumor therapeutics and contributing to advancing cancer treatment strategies.

## 2 Materials and methods

### 2.1 ACTL6A differential expression analysis

The upregulation of oncogenes in malignant tissues is a characteristic that sets malignancy unique (Eid et al., 2023). Consequently, in the preliminary phase of this investigation, we utilized data from TIMER2.0 (http://timer.cistrome.org/) (Li et al., 2020) to illustrate the disparities in ACTL6A gene expression between tumor and normal tissue. As normal tissue for comparison was not accessible in specific tumor models, we employed the Gene Expression Profiling Interactive Analysis 2 (GE-PIA2) database (http://gepia.cancer-pku.cn/) (Tang et al., 2019). Following that, a study was carried out to analyze the contrasting protein expression in cancerous and healthy tissue using the UALCAN program (https://ualcan.path.uab.edu/), which combines data from the Clinical Proteomic Tumor Analysis Consortium (CPTAC) (Chandrashekar et al., 2017). This work examined the levels of ACTL6A in normal, cancerous, and metastatic tissue using the TNMplot web server (https://tnmplot.com/analysis/). The purpose was to ascertain the gene expression’s connection with tumor growth (Bartha and Gyorffy, 2021).

### 2.2 Association between ACTL6A and tumor grade and stage

Tumor grade and tumor stage are pivotal parameters encompassing the assessment of tumor anomalies, including the evaluation of tumor size and invasion, respectively. These characteristics hold significant relevance in determining patient survival outcomes. To explore the potential correlation between ACTL6A and tumor stage and grade, we leveraged online resources, specifically the GEPIA2 and TISIDB platforms (Ru et al., 2019; Tang et al., 2019).

### 2.3 Assessment of differential ACTL6A protein levels

The UALCAN server (Chandrashekar et al., 2017) was deployed to analyze fluctuations in ACTL6A protein levels throughout a wide range of patient tumors. Immunohistochemistry (IHC) images depicting various proteins in both tumor and normal conditions were obtained from the HPA (https://www.proteinatlas.org/) (Uhlen et al., 2015). These images served as a validation resource to corroborate the outcomes generated by the UALCAN tool for the studied protein.

### 2.4 Survival prognosis analysis

To assess the predictive value of ACTL6A expression in various cancer types, we conducted a comprehensive analysis of survival prognosis. This analysis involved utilizing the GEPIA2 database and the KM plotter, accessible at https://kmplot.com/analysis/. Initially, the “Survival Analysis” module in GEPIA2 was deployed to generate a heatmap illustrating the relationship between ACTL6A expression and both overall survival (OS) and disease-free survival (DFS). OS refers to the duration from the diagnosis of a medical condition to death from any cause, while DFS encompasses the period from diagnosis to either disease recurrence or death from any cause. Subsequently, we corroborated and expanded upon our findings by utilizing the KM plotter (Lánczky and Győrffy, 2021) in five specific forms of cancer: breast, ovarian, lung, stomach, and liver malignancies. This facilitated a comprehensive statistical evaluation of the correlation between ACTL6A expression and patient survival. We considered potential confounding factors that could affect survival outcomes to verify the trustworthiness of our data. Adjusted analyses were conducted to improve the precision of the results by taking into consideration factors such as age, gender, treatment methods, and tumor stage. The meticulous methodology enhances the credibility of our findings about the predictive significance of ACTL6A expression, offering a full comprehension of its influence on OS and DFS in different types of cancers.

### 2.5 Connection between ACTL6A genetic alteration and patient survival

The progression of cancer is marked by a multitude of genetic alterations in various genes, notably those engaged in the regulation of normal cell growth. These anomalies lead to unregulated cell cycle advancement, ultimately resulting in the conversion of cells into malignant forms (Matthews et al., 2022). In this study, we employed the cBioPortal database (https://www.cbioportal.org/) (Gao et al., 2013) to scrutinize genetic alterations present in ACTL6A within tumor samples. The primary aim of our investigation was to evaluate the various types, locations, and impacts of ACTL6A mutations on clinical results.

### 2.6 Epigenetic alterations in ACTL6A within tumor microenvironment

The initiation of cancer frequently involves various epigenetic alterations that lead to the deactivation of genes restraining tumor growth and the activation of genes promoting cancer development (Qu et al., 2013). A pivotal epigenetic process in this context is DNA methylation (Tran et al., 2021). To explore the DNA methylation status of ACTL6A in tumor settings and make comparisons with normal controls, we employed two platforms: UALCAN (Chandrashekar et al., 2022) and SMART app (https://bio.tools/SMART_App#) (Li et al., 2019).

### 2.7 ACTL6A metamorphosis: exploring its impact on infiltration and functionality of varied immune components

A thorough investigation has been performed to explore the implications of the human immune system on tumor development, revealing the presence of diverse cells with varying effects (Smyth et al., 2006; Upadhyay et al., 2018). This study delves into the effects of ACTL6A genetic alterations on various components of the immune system within tumor environments. Recent studies have shown a strong connection between increased amounts of myeloid-derived suppressor cells (MDSCs) and negative outcomes, cancer advancement, and the immunotherapies’ efficacy. This correlation has been specifically noted in breast, colorectal, lung cancers, and hematologic malignancies (Solito et al., 2011; Yang Z. et al., 2020; Hao et al., 2021; Yu et al., 2022). Natural Killer T (NKT) cells possess the capability to eliminate target cells through both direct cytotoxicity (Metelitsa et al., 2001; Kuylenstierna et al., 2011) and by indirectly influencing immune cells coming from both myeloid and lymphoid lineages (McEwen-Smith et al., 2015). Hence, this investigation focuses on the MDSCs and NKT cell’s infiltration and status to discern any possible association with alterations in the ACTL6A gene. The investigation employed the TIMER2 web server (Li et al., 2020) to explore the correlation between ACTL6A alterations and the occurrence of CD8 T cell infiltration. Additionally, data from the SangerBox website were employed to investigate the correlation between ACTL6A expression in malignant tissue and microsatellite instability (MSI), tumor mutational burden (TMB), and immunological checkpoints (Shen et al., 2022).

### 2.8 Expression level of ACTL6A at the single-cell level

In assessing ACTL6A expression at the single-cell level within the TME, the TISCH (http://tisch.comp-genomics.org/) was employed (Han et al., 2023). To focus on untreated tumors, we intentionally excluded datasets involving treated, metastatic, and relapsed cases, aiming for a clearer baseline representation of ACTL6A expression. This selective exclusion, while ensuring a focused examination, does bring about potential limitations, primarily impacting the generalizability of our findings to diverse tumor stages and introducing a degree of bias. Our analysis using the “pheatmap” package in R provides a snapshot of ACTL6A expression dynamics within this context. Researchers should consider these limitations when interpreting the results.

### 2.9 ACTL6A enrichment analysis

In advance of conducting the enrichment analysis, we constructed two distinct sets of proteins: one consisting of ACTL6A interacting proteins and the other consisting of ACTL6A correlated proteins in the TME. The STRING (https://string-db.org/) (Szklarczyk et al., 2021) and GEPIA2 (Tang et al., 2019) databases were utilized to generate these lists, respectively. Subsequently, a Venn diagram (http://bioinformatics.psb.ugent.be/webtools/Venn/) illustrating the shared proteins between the two lists was constructed.

To bolster the precision of cancer detection through machine learning methodologies, we meticulously acquired LIHC gene expression data from The Cancer Genome Atlas (TCGA) (Weinstein et al., 2013), utilizing the TCGAbiolinks package within the R programming environment (Mounir et al., 2019). This comprehensive dataset comprised 50 normal samples and 371 primary tumor samples. To refine our analysis, we strategically extracted only the gene expression profiles of the genes that were common between STRING and GEPIA2 datasets. Employing a multifaceted approach, we applied four distinct machine learning models—logistic regression (LR), support vector machine (SVM), random forest (RF), and XGBoost. The performance of these models was systematically evaluated and compared using Receiver Operating Characteristic (ROC) curve analysis implemented through the Scikit-learn library in Python (Pedregosa et al., 2011).

In order to clarify the molecular pathways that contribute to the cancer-causing function of ACTL6A, we conducted an enrichment analysis on the proteins obtained from the two lists stated above. This analysis was performed using the DAVID (Sherman et al., 2022).

### 2.10 Molecular docking

**FIGURE 2:**
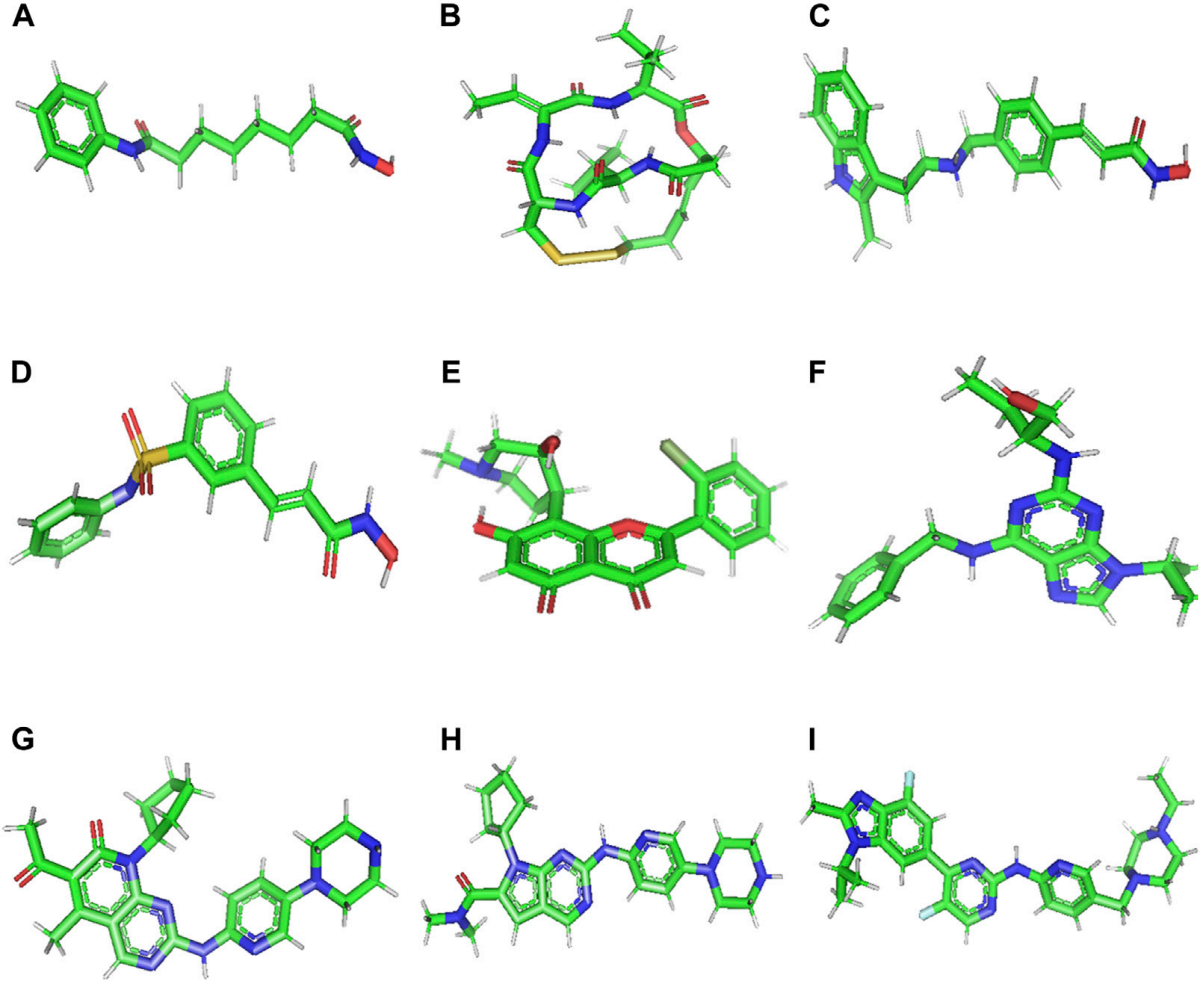
3D structure of Vorinostat **(A)**, Romidepsin **(B)**, Panobinostat **(C)**, Belinostat **(D)**, Flavopiridol **(E)**, Roscovitine **(F)**, Palbociclib **(G)**, Ribociclib **(H)**, and Abemaciclib **(I)**.

Accordingly, molecular docking was performed to assess the binding of 9 small molecules reported as potential anti-cancer agents against the ACTL6A target. Four histone deacetylase inhibitors, Vorinostat, Romidepsin, Panobinostat, and Belinostat, were selected as they were reported for their capability to reverse the effect of ACTL6A overexpression (Xiao et al., 2021). Also, pan-cyclin-dependent kinase inhibitors (CDK inhibitors) and antitumor activity effect are suggested to be mediated via ACTL6A suppression (Shrestha et al., 2020). Five pan-CDK inhibitors, flavopiridol, roscovitine, palbociclib, ribociclib, and abemaciclib, were utilized as potential hits in this study. In AutoDockTools (ADT, v1.5.6), the nine small molecules illustrated in Figure 2 were created. This involved the manipulation of the prepare_ligand4.py command, and the resulting structures were saved in PDBQT format.

The receptor structure was downloaded from the AlphaFold structure prediction website (https://alphafold.ebi.ac.uk/entry/O96019), which modeled the 3D structure of ACTL6A protein by modeling uniport entry (O96019 · ACL6A_HUMAN) on chain K of PDB ID. 7vdv as a template possessing a coverage of 78%. The structure was downloaded in PDB format and prepared using AutoDock Vina. Polar hydrogens were added, and energy was minimized by utilizing the prepare_receptor4.py command of the ADT. The partial atomic charge was calculated using Kollman-united charge, and the prepared file was saved in PDBQT format.

In the absence of reported binding pockets, structures were docked in all protein pockets to identify suitable binding sites. Employing the Lamarckian genetic algorithm, the docking parameters were set to 2,500,000 energy evaluations, 100 runs, and a population size of 150 (Morris et al., 1998; Ross, 2019). Ten binding variants were generated for the ligands, each exhibiting a maximum energy difference of 3 kcal/mol. The most favorable conformations, which indicate the lowest binding free energy, were retrieved. The software BIOVIA Discovery Studio Visualizer 2021 was utilized to generate visual representations of 2D interaction figures (Morris et al., 1998; Biovia, 2017; Ross, 2019).

In embarking on enrichment analysis, it’s vital to recognize its reliance on the thoroughness and precision of interaction data sourced from STRING and GEPIA2 databases. Assumptions tied to this analysis pivot on the availability of comprehensive and reliable data, raising awareness of potential biases arising from unevenly represented interactions. Caution in result interpretation is advised, with a clear understanding of inherent data limitations and their potential impact on enrichment outcomes.

### 2.11 Trichostatin A and Vorinostat at the ACTL6A active site as simulated using MD

MD simulation of 100 ns was applied for 3 best hits, one chosen for each pocket) on ACTL6A. Palbociclib, Ribociclib, and Romidepsin were chosen for Pockets A, C, and B, respectively. MD simulation was carried out using GROMACS-2023.1, where the protein topology was prepared using the CHARMM36 force field, whereas the ligand topology was prepared using the CGenFF server of the General force field. A dodecahedral unit cell box was used in the solvation process, and periodic boundary conditions with 10 Å were used. Ions were introduced using the steepest descent minimization algorithm, and sodium and chloride ions were used for protein neutralization. Energy minimization was applied to the complex to avoid steric clashes using the steepest descent minimization algorithm, force cutoff was set to 10.0 kJ/mol, and the maximum number of steps was 50,000. Next, 2 equilibration processes were applied: NVT and NPT equilibration using a modified Berendsen thermostat and leap-frog integrator for 50,000 steps equivalent to 10 ps. Finally, the MD simulation run for 50 ns with 2 fs at each step. The Molecular Mechanics Poisson Boltzmann Surface Area (MM/PBSA) via gmx_mmpbsa (Homeyer and Gohlke, 2012) was used for binding free energies calculations, calculating the relative binding free energies, non-polar solvation energy, according to the following equation (Eqs 1–4):

ΔGbind,aq=ΔH − TΔS≈ΔEMM+ΔGbind,solv − TΔS (1)

ΔEMM=ΔEcovalent+ΔEelectrostatic+ΔEvdW (2)

ΔEcovalent=ΔEbond+ΔEangle+ΔEtorsion (3)

ΔGbind,solv=ΔGpolar+ΔGnon−polar (4)

In the given equation, ΔEMM reflects the change in energy of the gas-phase molecular mechanics (MM), ΔGbind, solv indicates the change in free energy due to solvation, and − TΔS relates to the change in conformational entropy during binding. The computation of these changes is achieved by performing ensemble averaging over a substantial collection of sampled conformations.ΔEMM is determined by three components derived from MM: the alteration in covalent energy (ΔEcovalent), the modification in electrostatic energy (ΔEelectrostatic), and the variation in van der Waals energy (ΔEvdW). ΔEcovalent encompasses alterations in the bond terms (ΔEbond), the angle terms (ΔEangle), and the torsion terms (ΔEtorsion). The solvation-free energy change (ΔG-bind, solv) is commonly decomposed into polar and non-polar components (ΔGpolar and ΔGnon-polar).

## 3 Results

### 3.1 ACTL6A is overexpressed in several human tumors compared to normal tissue

**FIGURE 3:**
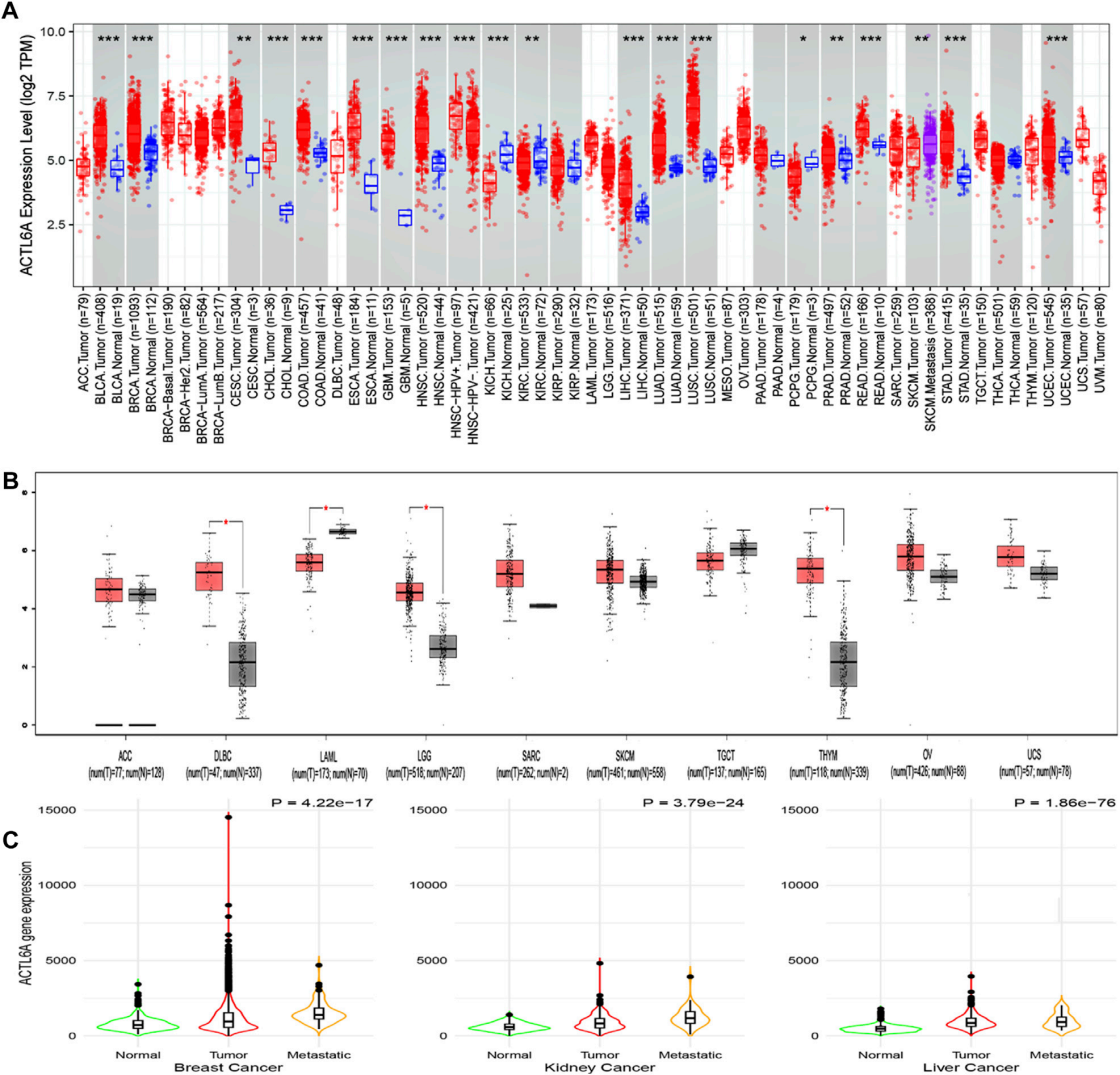
Analysis of ACTL6A expression in human cancers. **(A)**: The expression of ACTL6A was analyzed in a variety of TCGA cancers using TIMER2.0 to determine differential expression **(B)**: GEPIA2 database was employed to assess cancers lacking equivalent normal tissue for comparison. These tumors demonstrated a progressive rise in ACTL6A expression in contrast to normal tissue that was validated by the GEPIA database **(C)**: Tumors repeatedly showed a clear link between the expression of ACTL6A and the kind of tissue, specifically in the order of normal, malignant, and metastatic tissue.

This work employed TIMER2 to investigate the disparities in ACTL6A expression levels between malignant and normal tissues. The expression of ACTL6A was shown to be significantly increased in many forms of cancer, encompassing BLCA, BRCA, CHOL, COAD, ESCA, GBM, HNSC, LIHC, LUAD, LUSC, READ, STAD, and UCEC (*p* < 0.001). Additionally, in CESC and PRAD, the overexpression of ACTL6A was also statistically significant (*p* < 0.01, Figure 3A). Due to the lack of normal tissue samples for comparison in 10 tumors. ACTL6A was shown to be significantly overexpressed in three tumors, namely DLBC, LGG, and THYM, according to data obtained from the GEPIA2 database (*p* < 0.05, Figure 3B). No statistically significant modifications were observed in six kinds of cancer, namely ACC, SARC, SKCM, TGCT, OV, and UCS. However, in the case of LAML, there was a notable increase in the expression of ACTL6A in normal tissues compared to malignant tissues. The current study employed the “compare tumor, normal, and metastasis” module of the TNMplot web server to examine the correlation between ACTL6A mRNA expression levels and the incidence and spread of cancer. Figure 3C exhibits a substantial increase in ACTL6A expression in tumor tissues compared to normal tissues in several types of cancers, such as breast, kidney, and liver. The enhanced manifestation of ACTL6A is still apparent when comparing the ACTL6A expression in tumor and metastatic tissues.

### 3.2 The association between ACTL6A expression and tumor stage and grade in multiple malignancies in humans

**FIGURE 4:**
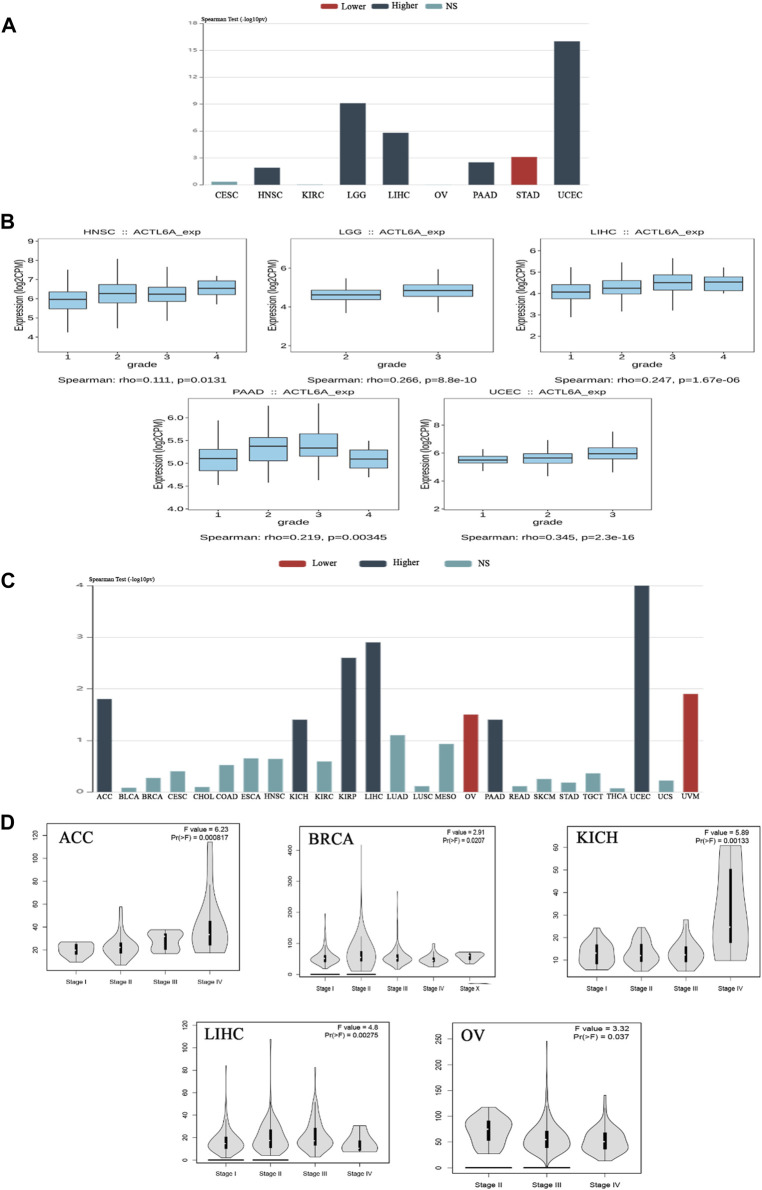
The association between the expression of ACTL6A and the stage and grade of the tumor. **(A)**: A bar graph demonstrates the relationship between ACTL6A expression and tumor grade **(B)**: A box plot exhibits a direct link between ACTL6A levels and tumor grade **(C)**: A bar graph illustrating the correlation between ACTL6A expression levels and tumor stage **(D)**: The violin plot indicates a positive correlation between the level of ACTL6A and the stage of the tumor.

Upon verifying the ACTL6A upregulation at mRNA and protein levels, we aimed to examine the potential consequences of this overexpression on the severity and progression of human malignancies. The examination of the TISIDB web server data manifested a significant association between ACTL6A expression as well as the tumor grade in HNSC, LGG, LIHC, PAAD, and UCEC (*p* < 0.001, Figures 4A, B). The TISIDB web server research illustrated a significant connection between ACTL6A expression and six distinct tumor types within the tumor stage framework, specifically ACC, KICH, KIRP, LIHC, PAAD, and UCEC (Figure 4C). In a similar direction, the analysis of the GEPIA2 database depicted a positive association between ACTL6A expression and these cancer types: ACC, BRCA, KICH, LIHC, and OV (Figure 4D). The comparative analysis of the two databases showed significant findings on the association between the ACTL6A level and tumor stage in three distinct types of malignancies, particularly ACC, KICH, and LIHC.

### 3.3 Assessment of differential ACTL6A protein levels

**FIGURE 5:**
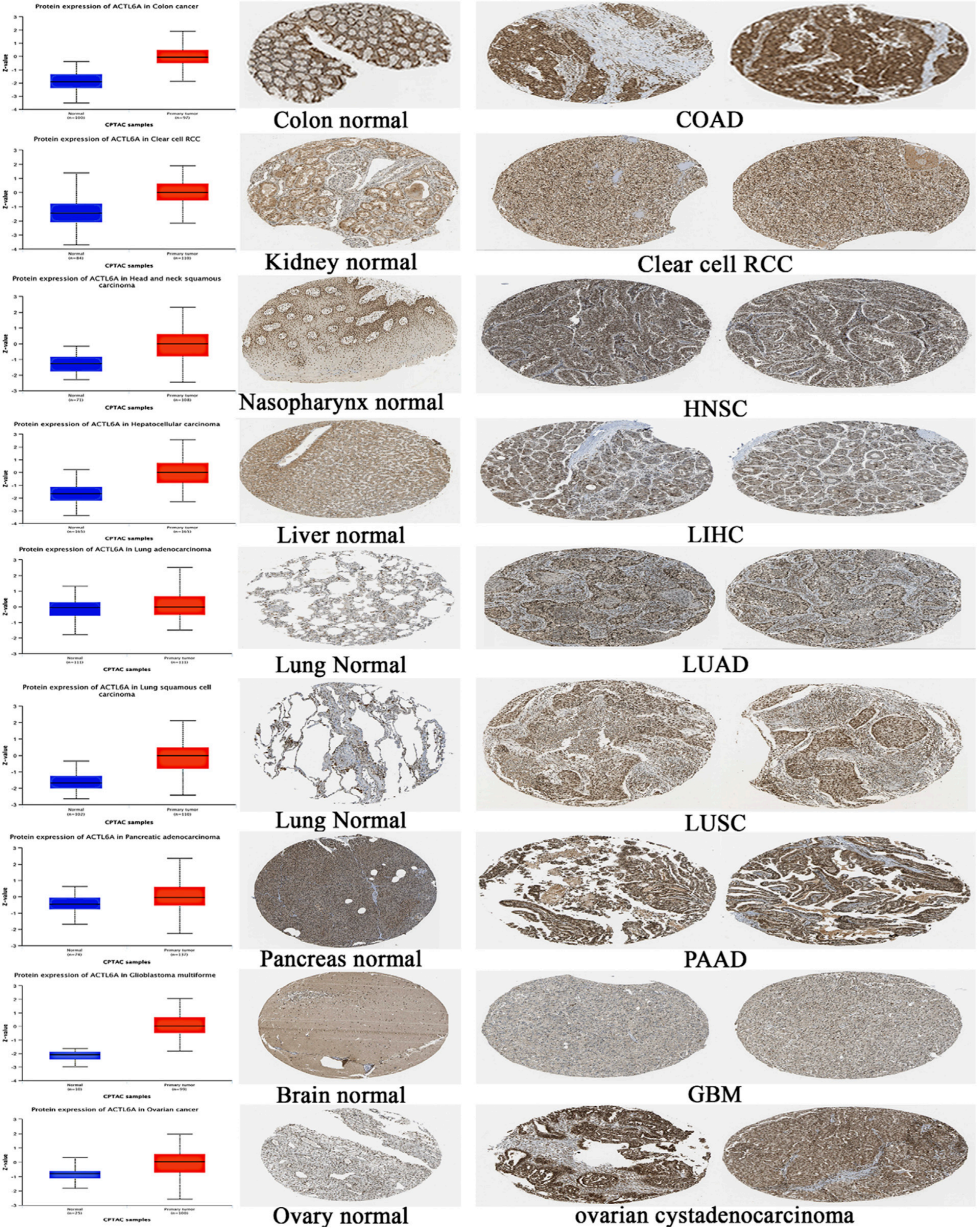
A statistically significant rise in ACTL6A protein expression in tumor samples in comparison to normal samples (on the left side). The IHC staining confirmed the same results, showing a steady increase in both normal tissue (center) and malignant tissue (right).

After analyzing the ACTL6A gene at the level of gene expression, we then assessed its protein expression by utilizing the extensive proteome data supplied by the National Cancer Institute’s CPTAC dataset. The results of this study demonstrate a notable elevation in the expression of ACTL6A protein in tumor tissues of the colon, clear cell RCC, HNSC, HCC, LUAD, LUSC, PAAD, GBM, and OV in comparison to normal tissues. The statistical analysis provided evidence for this discovery, as indicated by *p*-values less than 0.05 (Figure 5). Following that, IHC Figures were acquired for both normal and malignant tissues to authenticate our previous findings. The results revealed a consistent pattern, with the staining intensity ranging from low to intermediate in the normal tissues of the colon, kidney, nasopharynx, liver, lung, pancreas, brain, and ovary. On the other hand, the staining intensity in the malignant tissues was found to be moderate to high (Figure 5).

### 3.4 The inverse relationship between elevated ACTL6A levels and clinical outcomes

**FIGURE 6:**
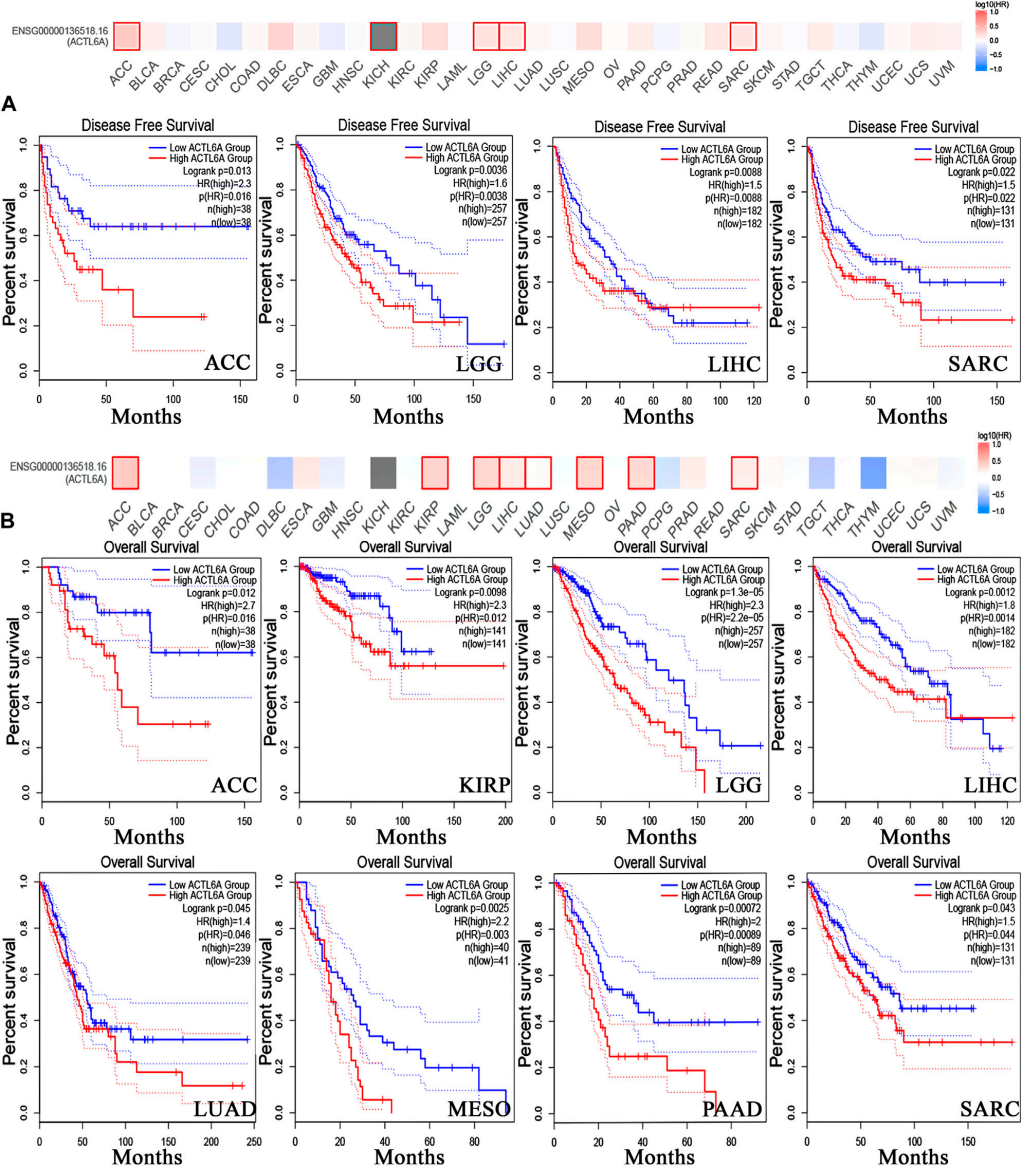
Relationship between clinical outcome and expression of ACTL6A. According to the GEPIA2 database, two important factors are considered. **(A)**: Disease free survival **(B)**: the overall survival. Tumors that have a significant correlation are depicted in boxes.

**FIGURE 7:**
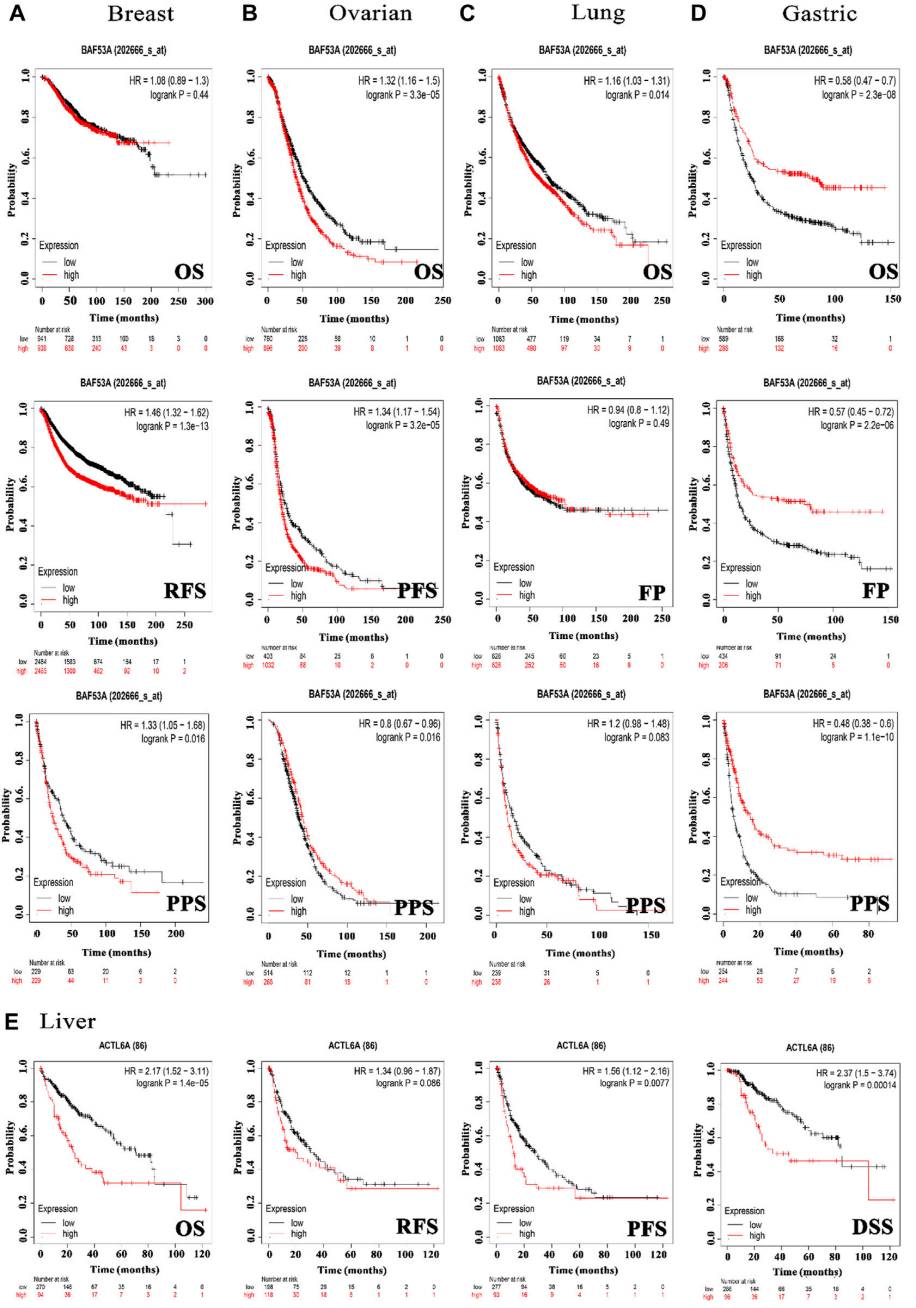
The association between ACTL6A expression and survival prognosis was assessed employing the KM plotter tool for multiple types of cancer, namely **(A)** breast, **(B)** ovarian, **(C)** lung, **(D)** gastric, as well as **(E)** liver cancer.

In order to investigate the correlation between ACTL6A expression and patients’ survival, we utilized two datasets, namely GEPIA and KM plotter. The analysis of data from the GE-PIA database indicated a strong correlation between the expression of the target gene and a negative prognosis for ACC, LGG, LIHC, and SARC (*p* < 0.05) in terms of DFS (Figure 6A). Nevertheless, the analysis of patients’ OS indicated that individuals with PAAD (*p* < 0.001), ACC, KIRP, LGG, LIHC, LUAD, MESO, and SARC (*p* < 0.05) had an unfavorable prognosis (Figure 6B). The KM plotting analysis revealed a negative connection between breast cancer and both RFS and PPS, as shown in Figure 7. In contrast, ovarian cancer exhibited a negative connection with all of the investigated parameters. Furthermore, lung cancer possesses a sole adverse correlation in terms of OS. Gastric tumors exhibited a negative link between OS and disease-free progression (FP). ACTL6A expression in liver tumors showed a negative correlation with patient survival in terms of progression-free survival (PFS) and disease-specific survival (DSS), as illustrated in Figure 7.

### 3.5 The correlation between genetic alteration and patient outcome, specifically focusing on predicting poor prognosis

**FIGURE 8:**
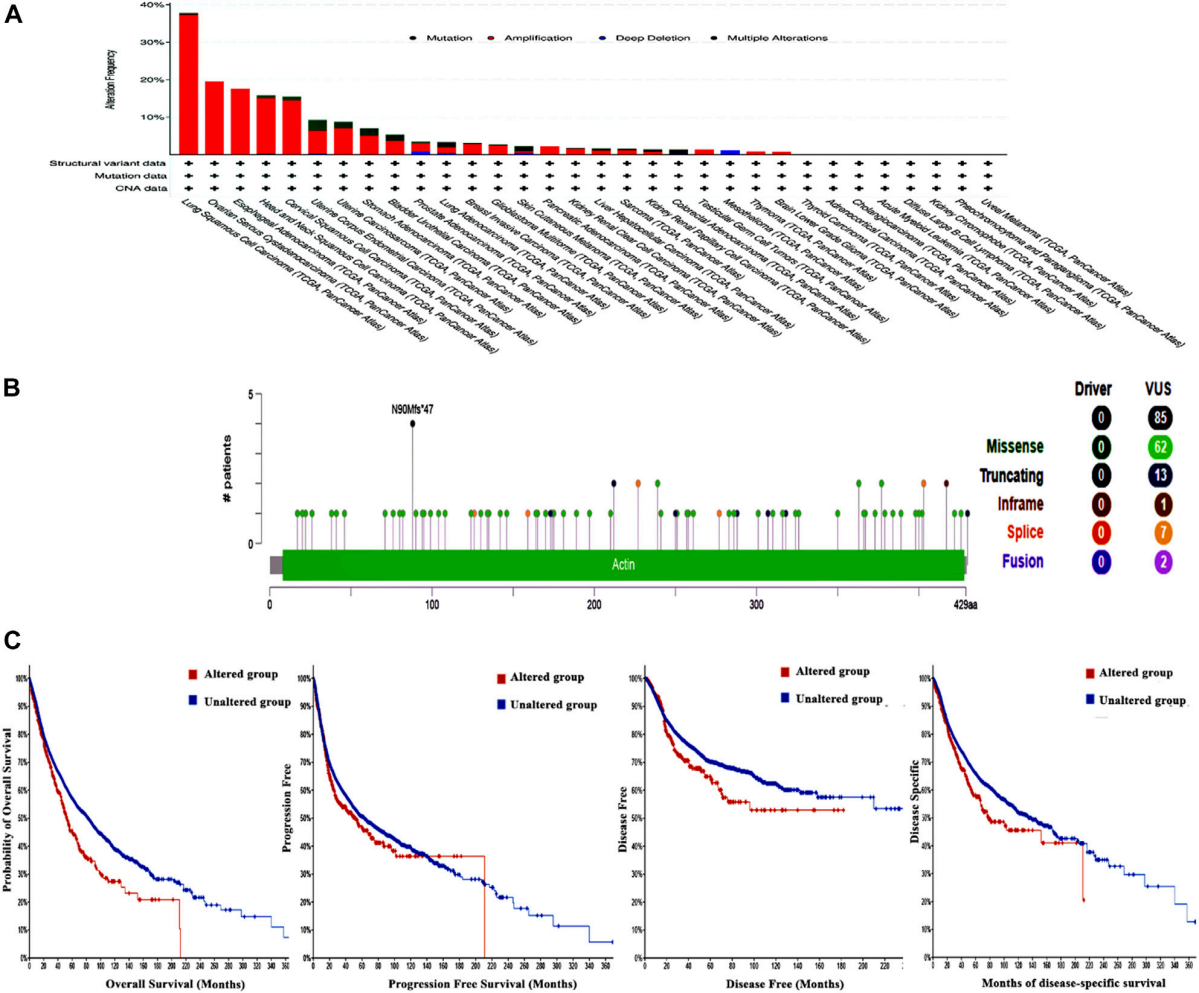
Assessment of ACTL6A gene mutations using the cBioPortal program **(A)**: Conducting a frequency analysis to examine the variations in mutation types throughout a human tumors panel that are currently being investigated **(B)**: A map illustrating the positions and categories of ACTL6A mutations **(C)**: The correlation between ACTL6A mutations and the absence of disease, the absence of disease-specific to a certain condition, the absence of disease progression, and OS status.

The examination of the cBioPortal database produced findings that suggest lung squamous cell carcinoma exhibits the most significant occurrence of genetic mutations in ACTL6A among all types of human malignancies, with an approximate change frequency of 39%. In addition, the majority of human malignancies analyzed revealed that “amplification” was the primary form of genetic modification, except for colorectal adenocarcinoma and mesothelioma, in which mutation was identified as the predominant ACTL6A genetic alteration for colorectal adenocarcinoma and deep deletion for mesothelioma (Figure 8A). The analysis of the ACTL6A mutation variants indicated that the prevailing form was the missense mutation. Additionally, significant modifications were identified at site N90 MF in ACTL6A (Figure 8B). Regarding the analysis of genetic anomalies in ACTL6A, it was noted that in two out of four models being studied, namely overall survival and disease-free, a significant negative correlation was identified between ACTL6A mutations and patient survival (Figure 8C).

### 3.6 The divergent methylation patterns of ACTL6A in various human cancers

**FIGURE 9:**
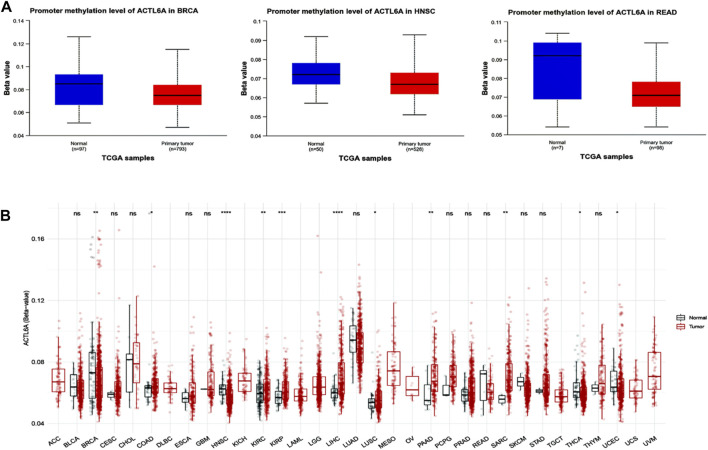
Performing differential methylation study on ACTL6A in tumor samples as compared to normal samples. **(A)**: The UALCAN analysis revealed that the ACTL6A promoter region had a higher degree of methylation in tumors compared to normal tissues **(B)**: Analyzing the methylation of ACTL6A in a database of human tumors employing a method that aggregates CpG sites. The notation for statistical significance is as follows: *p* > 0.05 is manifested by “ns,” *p* ≤ 0.05 by “, *p* ≤ 0.01 by”, “*p* ≤ 0.001 by”, “and *p* ≤ 0.0001 by ”****.

Significant results were obtained from the methylation evaluation conducted using the UALCAN web server. The investigation represented that two types of tumors, HNSC and READ, demonstrated a state of promoter hypomethylation compared with normal samples (*p* < 0.05, Figure 9A). Furthermore, the tumor types BRCA exhibited the same pattern of promoter hypomethylation, with statistical significance (*p* < 0.01). Moreover, the results obtained from the SMART program revealed that BRCA, HNSC, THCA, and UCEC exhibited a reduction in CpG-aggregated methylation levels compared with their corresponding normal counterparts (Figure 9B).

### 3.7 A positive link was seen between ACTL6A expression of ACTL6A in malignant tissue and the presence of immunosuppressive cells

**FIGURE 10:**
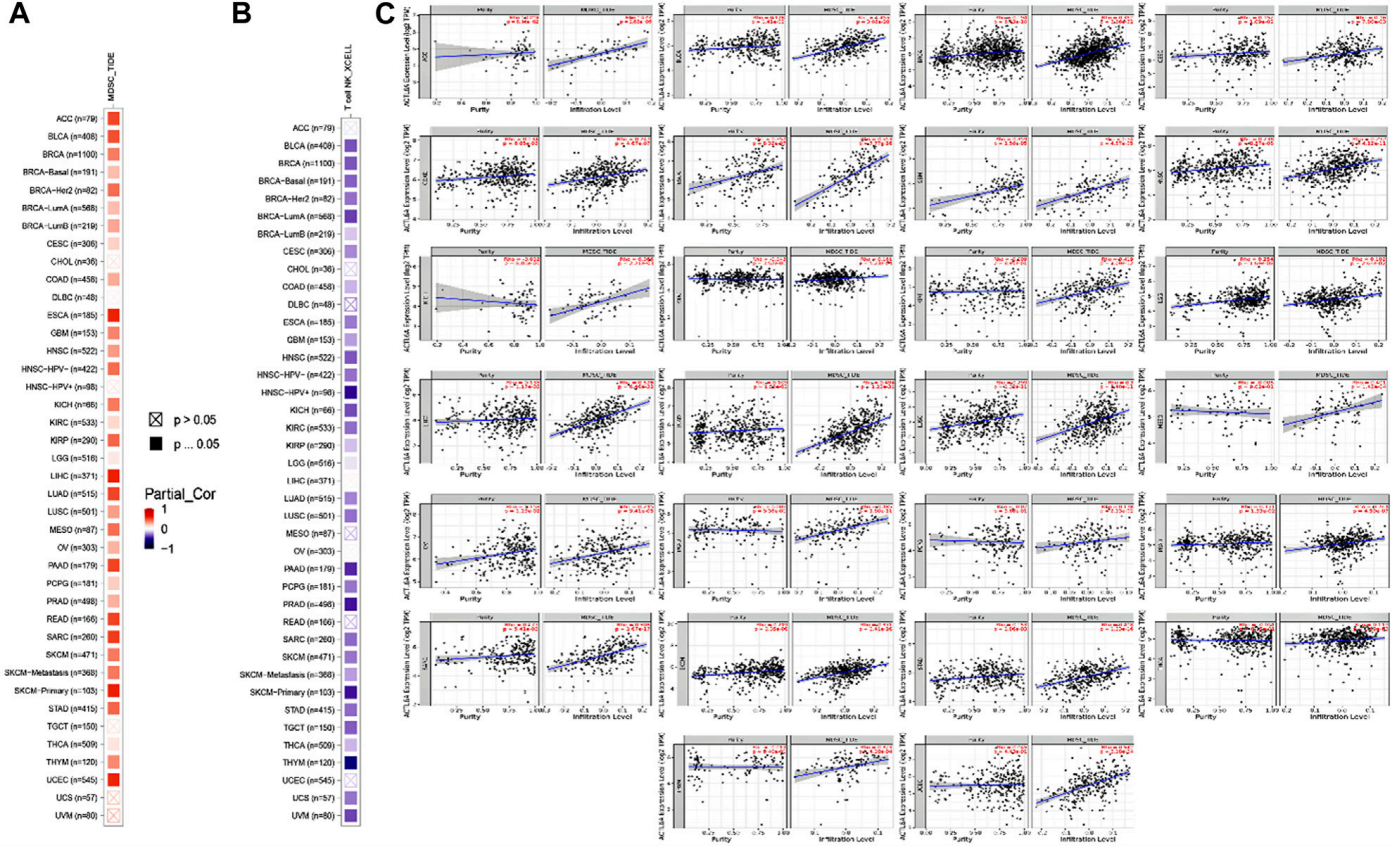
The correlation between ACTL6A expression levels and the presence of **(A)**: MDSC **(B)**: NKT cells in different types of human malignancies **(C)**: Scatter plots depict the correlation between ACTL6A expression and the level of MDSC invasion.

**FIGURE 11:**
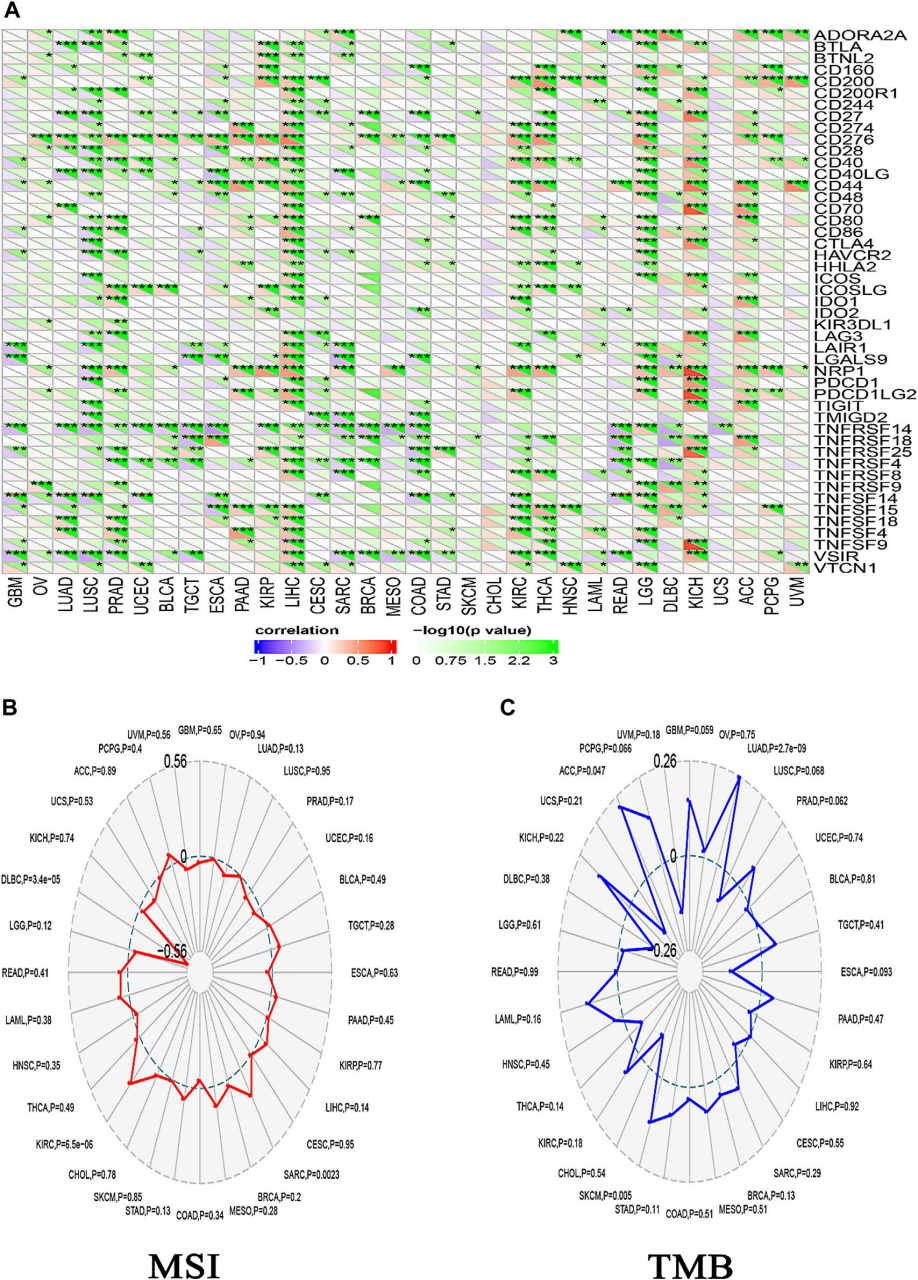
The relationship between ACTL6A expression, immunological checkpoints, MSI, and TMB is being examined. **(A)**: A heatmap illustrates the correlation between immune checkpoints and ACTL6A in various human malignancies **(B,C)**: The ordered pair Radar charts display the intersections of ACTL6A with MSI and TMB correspondingly. (*: *p*-value <0.05; **: *p*-value <0.01; ***: *p*-value <0.001).

Different immune cell types with specific functions have been confirmed in tumor environments. This research primarily examined two distinct cell types: MDSC, which has immunosuppressive functions in cancer, to investigate any possible association between ACTL6A expression and malignant tissue (Gabrilovich and Nagaraj, 2009), and NKT, known for its different antitumor properties. Regarding MDSC, a substantial number of the tumors analyzed exhibited a notable and statistically significant association between the levels of ACTL6A and MDSC in many tumors. It is important to highlight that within the investigated panel, no tumor exhibited a negative connection between ACTL6A expression and MDSC invasion (Figures 10A–C). On the contrary, a negative connection was observed for NKT cells in most of the examined tumors (Figure 10B). Collective examination of the data revealed that BLCA, BRCA, CESC, COAD, ESCA, GBM, HNSC, KICH, KIRC, KIRP, LGG, LUAD, LUSC, PCPG, PRAD, SKCM, STAD, and THCA demonstrated a positive significant association between ACTL6A and MDSC and a negative association between ACTL6A and NKT cells. The output from the SangerBox web server showed that LGG, KIRC, and LIHC experienced a positive correlation between ACTL6A and the expression of several immune checkpoints. In contrast, LUSC showed a negative correlation with most of the immune checkpoints (Figure 11A). Moreover, tumors DLBC, KIRC, and SARC demonstrated a positive correlation between the ACTL6A and the MSI (Figure 11B). Finally, two tumors, namely LUAD and SKCM, experienced a significantly positive correlation between ACTL6A and the TBM (Figure 11C).

### 3.8 SC analysis of ACTL6A in cancers

**FIGURE 12:**
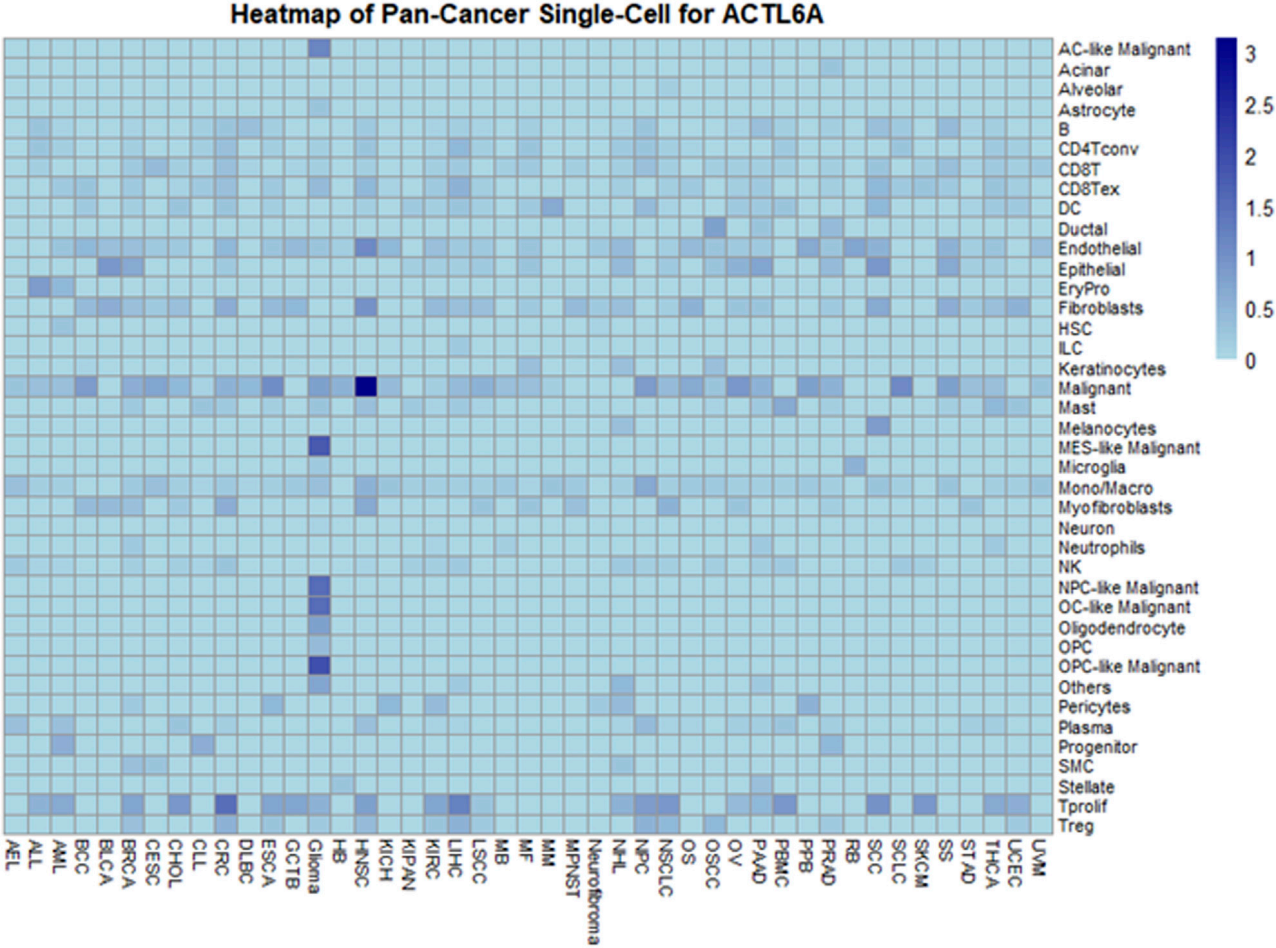
Comprehensive Single-Cell Analysis of ACTL6A across multiple cancer types.

In exploring ACTL6A expression at the single-cell level within the TME using the TISCH, a comprehensive single-cell (SC) analysis was conducted across 136 datasets spanning 45 cancer types. The resulting heatmap, depicted in Figure 12, provides insights into ACTL6A expression across 40 distinct cell types, including immune, stromal, malignant, and functional cells. Our findings indicate that ACTL6A expression is notably prominent in immune cells, particularly in monocyte/macrophage populations. Additionally, significant expression is observed in malignant cells. This comprehensive analysis sheds light on the preferential expression of ACTL6A in specific cell types within the TME, contributing to a nuanced understanding of its role in various cellular contexts.

However, it's essential to interpret these results in the context of the exclusion criteria applied during data analysis, which focused on untreated tumors. This strategic exclusion may influence the observed distribution of ACTL6A expression across cell types and warrants consideration when generalizing these findings to diverse tumor stages and treatment conditions. Future research, encompassing a broader spectrum of tumor contexts, will be pivotal for refining and expanding upon these observed expression patterns.

### 3.9 Analysis of interacting and correlated proteins to ACTL6A

ACTL6A has a notable association with the survival of cancer patients and has an implication on immune cells in the TME. Therefore, it is essential to ascertain the possible molecular pathways linked to this gene in different kinds of malignancies.

**FIGURE 13:**
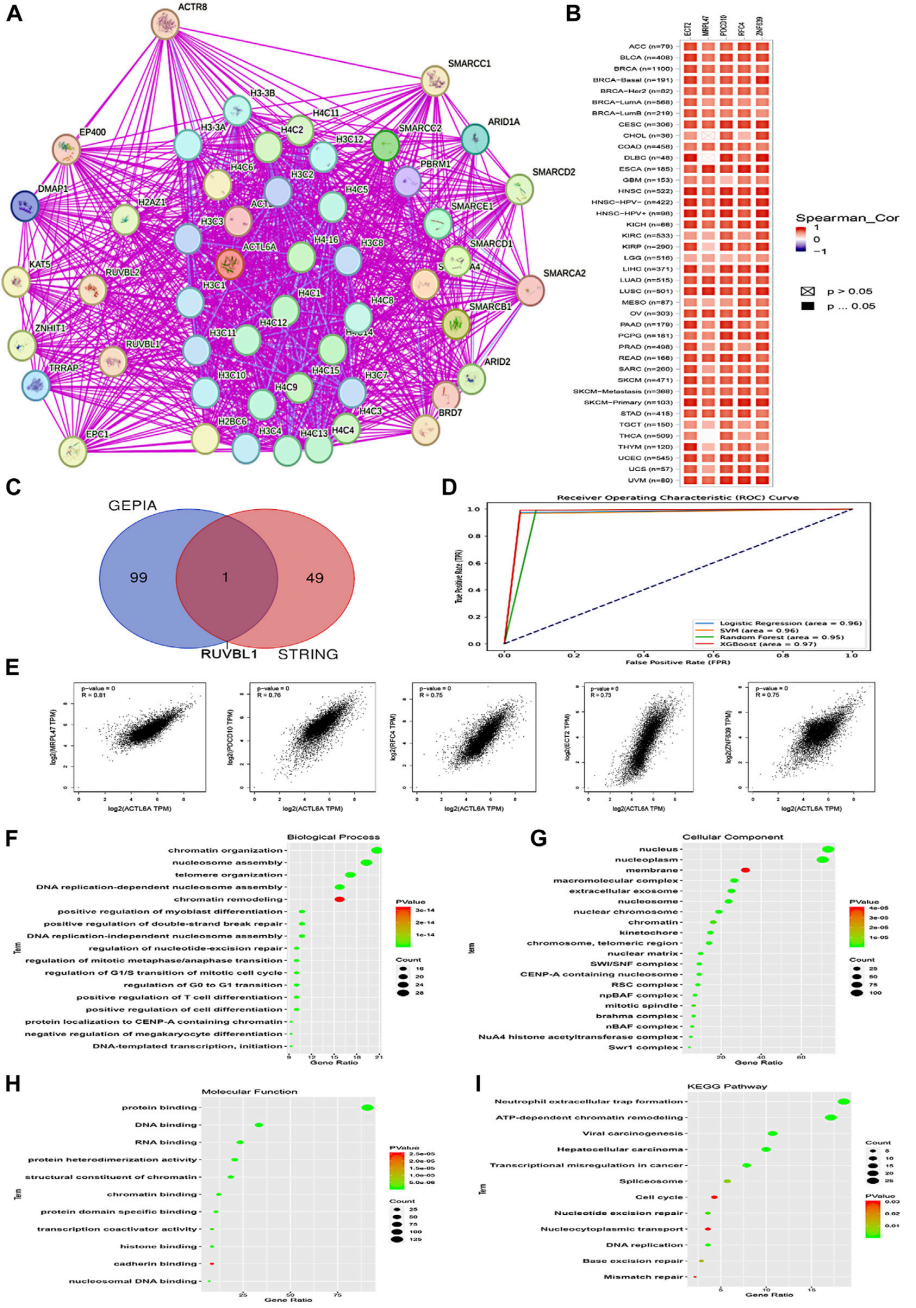
Interactions within the ACTL6A protein network. **(A)**: The STRING database has identified the top 50 proteins that interact with ACTL6A, and these interactions are illustrated on a map **(B)**: A heatmap visually displays the expression levels of the top five proteins linked to ACTL6A in tumor tissue **(C)**: A Venn diagram provides a visual representation of the proteins that have both interactions and correlations with ACTL6A **(D)**: ROC curve for AUC of LR, SVM, RF, and XGboost **(E)**: The GEPIA2 tool was used to investigate the link between ACTL6A expression and other genes (MRPL47, PDCD10, RFC4, ECT2, ZNF639) **(F-I)**: The enrichment study employed the KEGG and GO databases, with a specific focus on genes that demonstrate binding affinity to ACTL6A and interact with ACTL6A.

To begin, the top 50 experimentally validated that ACTL6A-interacting proteins were extracted from the STRING database, resulting in the construction of a protein-protein interaction network (Figure 13A). Furthermore, we used the GEPIA2 webserver to detect 100 genes that are linked to ACTL6A in the TCGA tumor panel. In our investigation, we deployed the “Correlation Analysis” module to produce plots that demonstrate the top five genes with the highest correlation. These genes include MRPL47 (R = 0.81), PDCD10 (R = 0.76), RFC4 (R = 0.75), ECT2 (R = 0.73), and ZNF639 (R = 0.75) (Figure 13E). Furthermore, the “Gene Corr” module at TIMER produced a heatmap that verified a strong positive link between these five genes and ACTL6A across all TCGA tumors (Figure 13B).

In our investigation, we found that ACTL6A has the most significant results in LIHC. We employed ACTL6A-related gene expression profiles for the detection of LIHC, and four machine learning models were employed—LR, SVM, RF, and XGBoost. The models demonstrated robust predictive performance, as reflected in their respective AUC values: LR (AUC = 0.96), SVM (AUC = 0.96), RF (AUC = 0.95), and XGBoost (AUC = 0.97). These results signify a high level of accuracy in distinguishing between normal and primary tumor samples. The ROC curve analysis visually illustrates the models’ superior discrimination ability, with XGBoost exhibiting the highest overall performance. The strong performance across all models suggests their potential utility in enhancing precision for cancer detection in the context of ACTL6A-associated protein expression patterns (Figure 13D).

After comparing two preexisting lists, it was determined that one gene, namely RUVBL1, had been duplicated (Figure 13C). After duplicates were eliminated, the two lists were merged in order to produce a distinct dataset. The dataset was then subjected to analysis utilizing the DAVID tool to enrich Reactome and Gene Ontology (GO) entriesThe analysis of biological processes revealed associations with various cellular activities, including chromatin organization, nucleosome assembly, telomere organization, DNA replication-dependent nucleosome assembly, chromatin remodeling, positive regulation of myoblast differentiation, positive regulation of double-strand break repair, DNA replication-independent nucleosome assembly, regulation of nucleotide-excision repair, regulation of mitotic metaphase/anaphase transition, regulation of G1/S transition of mitotic cell cycle, regulation of G0 to G1 transition, positive regulation of T cell differentiation, positive regulation of cell differentiation, protein localization to CENP-A containing chromatin, negative regulation of megakaryocyte differentiation, and DNA-templated transcription initiation (Figure 13F). Moreover, most genes were localized nuclei and nucleoplasm concerning cellular components. Membrane, macromolecular complex, extracellular exosome. Nucleosome, nuclear chromosome, chromatin, kinetochore, chromosome, telomeric region, nuclear matrix, SWI/SNF complex, CENP-A containing nucleosome, RSC complex, npBAF complex, mitotic spindle, Brahma complex, nBAF complex, NuA4 histone acetyltransferase complex, Swr1 complex (Figure 13G). Finally, regarding molecular function, the gene list showed enrichment for a range of functions, such as protein binding, DNA binding, RNA binding, protein heterodimerization activity, structural constituent of chromatin, chromatin binding, protein domain-specific binding, transcription coactivator activity, histone binding, cadherin binding, and nucleosomal DNA binding (Figure 13H). The enriched KEGG pathways encompassed Neutrophil extracellular trap formation, ATP-dependent chromatin remodeling, Viral carcinogenesis, Hepatocellular carcinoma, Transcriptional misregulation in cancer, Spliceosome, Cell cycle, Nucleotide excision repair, Nucleocytoplasmic transport, DNA replication, Base excision repair, Mismatch repair (Figure 13I).

### 3.10 Molecular docking

**FIGURE 14:**
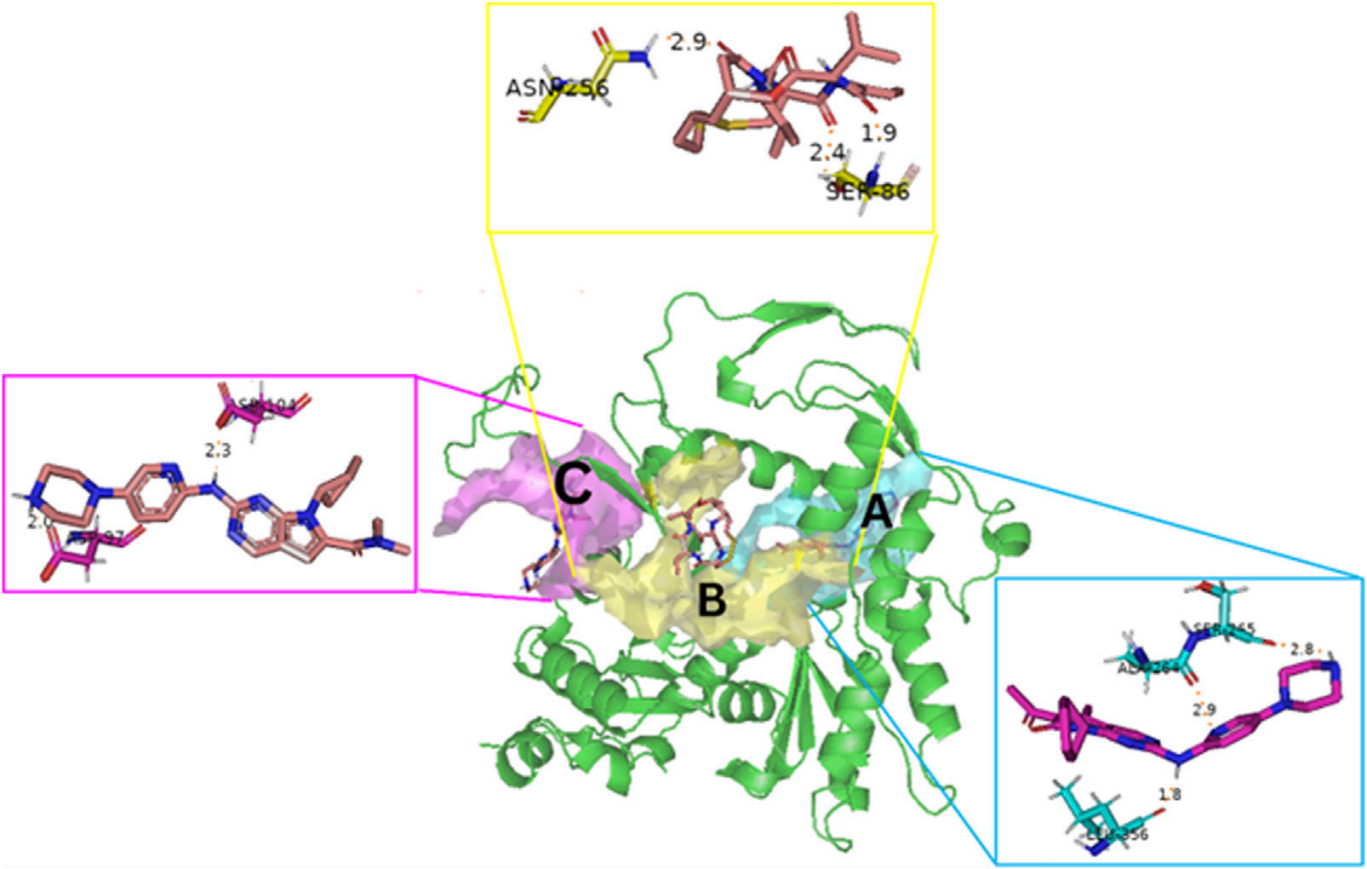
The 3D structure of the ACTL6A target shows the 3 potential reported binding pockets **(A)**: Pocket A in blue **(B)**: Pocket B in yellow **(C)**: Pocket C in magenta.

Blind docking of the 9 chosen small molecules against the ACTL6A receptor has suggested very good interaction and binding affinities for all the small molecules (−4.51 < ∆G < −8.09 kcal/mol), as shown in Table 1. They were able to bind to 3 major pockets: Pocket A, Pocket B, and Pocket C (Figure 14), where Palbociclib, Belinostat, and Roscovitine bound to Pocket A with binding energies of −8.09, −6.62, and −4.92 kcal/mol, respectively. Also, variable inhibition constants were *in silico* predicted for them: 1.18, 13.95, and 248.08 µM for Palbociclib, Belinostat, and Roscovitine, respectively. Belinostat, the only histone deacetylase inhibitor capable of binding to pocket A, was able to form 2 H-bonds with Gln263 and Arg390 in addition to a Pi-lone pair interaction with Asn 354, showing the best binding affinity among all the docked structures. Also, each of Palbociclib and Roscovitine managed to form 2 Honds and a Pi-lone pair interaction with Pocket A active residues. However, Belinostat and Roscovitine showed fewer binding affinities with higher predicted inhibition constants, about 14- and 250-fold, compared to Palbociclib.

**TABLE 1:** Overview of compounds: Bonded residues, 2D interactions, binding energies, and inhibition constants.

Cpd	Binding energy (Kcal/mol)	Inhib. Const. (µM)	Bonded residues	Type of interaction	2D interaction
Vorinostat Pocket C	−4.51	498.11	Lys62	H-bond	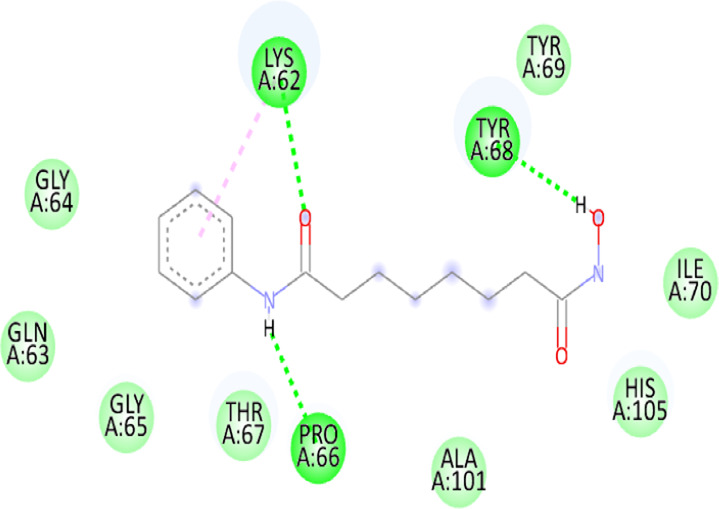
			Pro66	H-bond	
			Tyr68	H-bond	
Romidepsin	−6.52	16.49	Ser86	2 H-bond	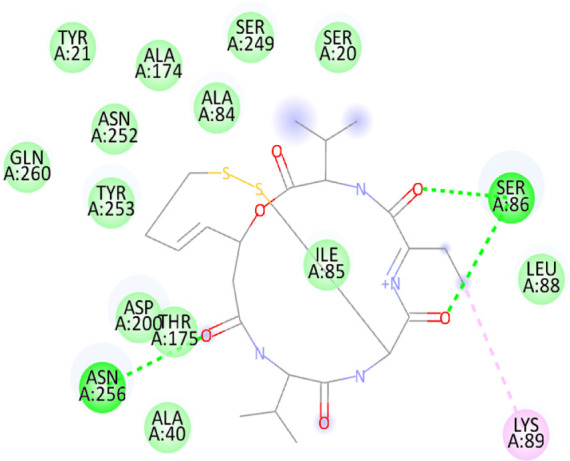
Pocket B			Asn256	H-bonds	
Panobinostat	−6.49	17.56	Ser86	H-bond	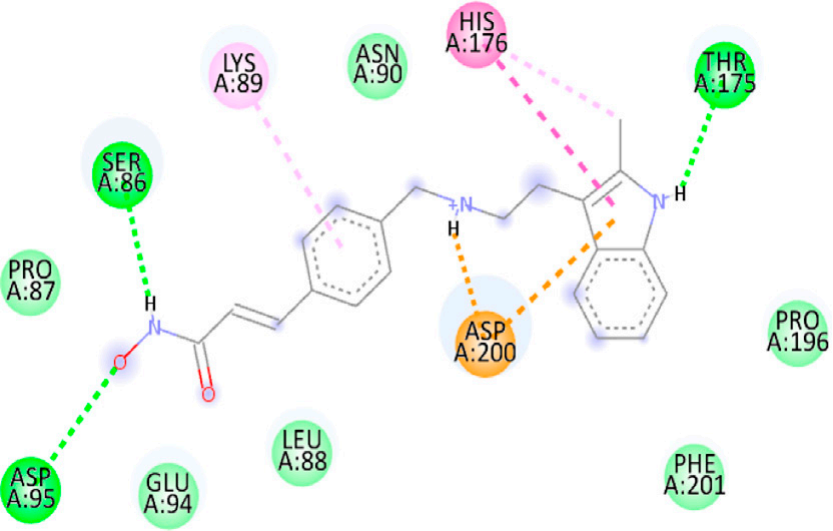
Pocket B			Asp95	H-bond	
			Thr175	H-bond	
			His176	Pi-Pi	
			Asp200	2 Pi-anion	
Belinostat	−6.62	13.95	Gln263	H-bond	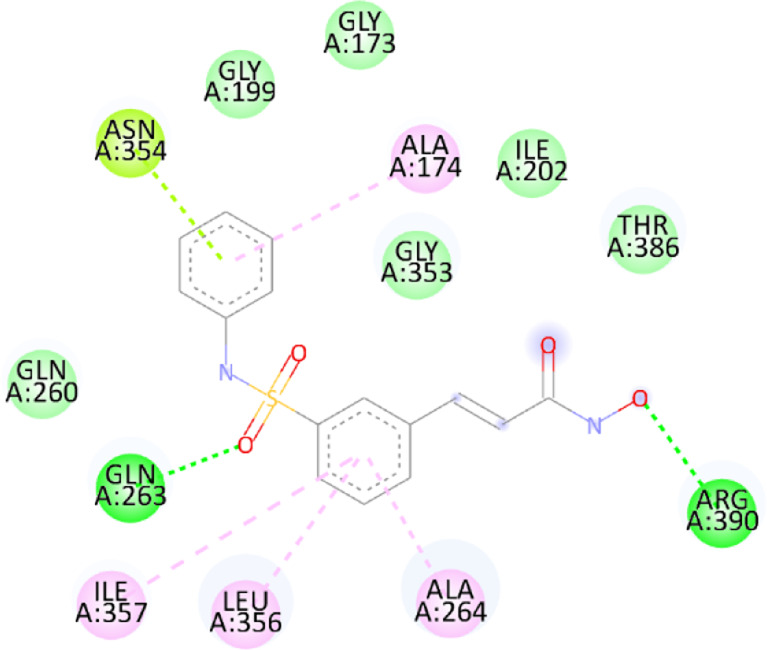
Pocket A			Asn354	Pi-lone	
			Arg390	H-bond	
Flavopiridol	−5.92	45.7	Lys62	Pi-cation	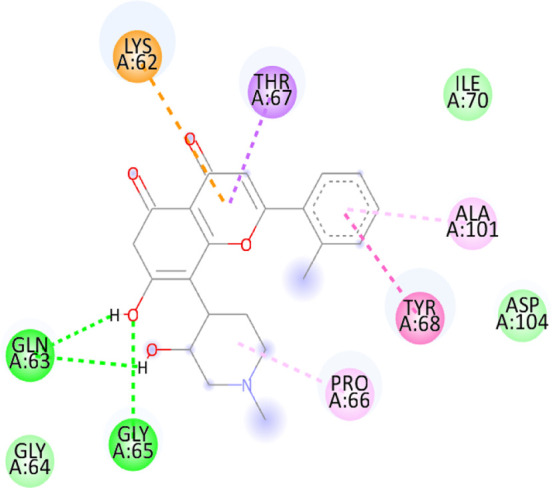
Pocket C			Gln63	2 H-bond	
			Gly65	H-bond	
			Thr67	Pi-sigma	
			Tyr68	Pi-Pi	
Roscovitine	−4.92	248.08	Tyr21	H-bond	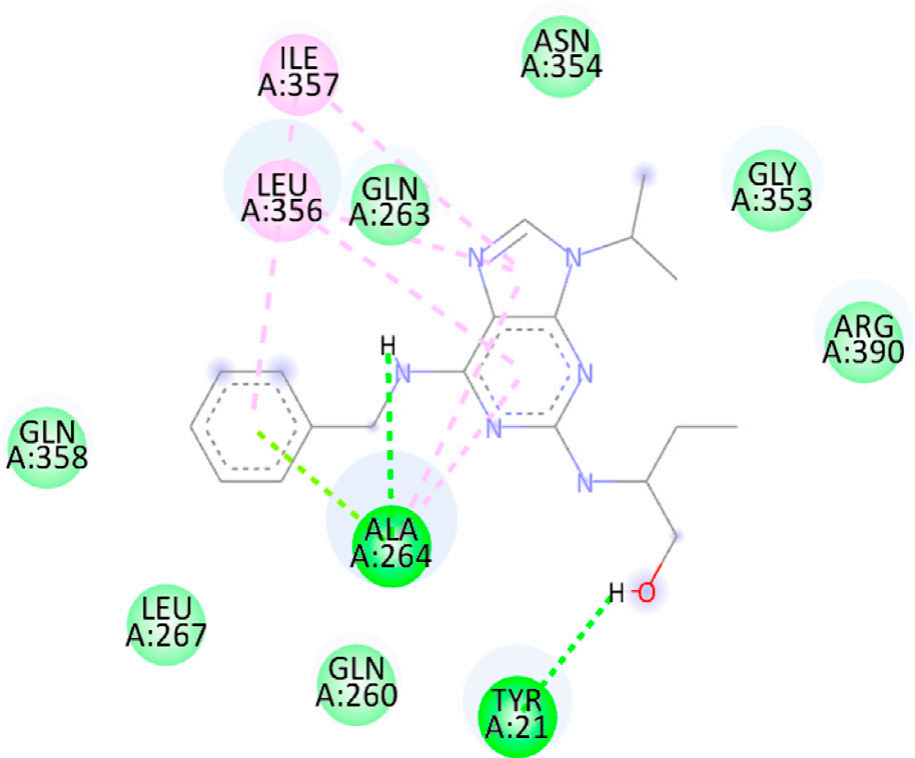
Pocket A			Ala264	H-bod + Pi-lone	
Palbociclib	−8.09	1.18	Ala264	Pi-lone	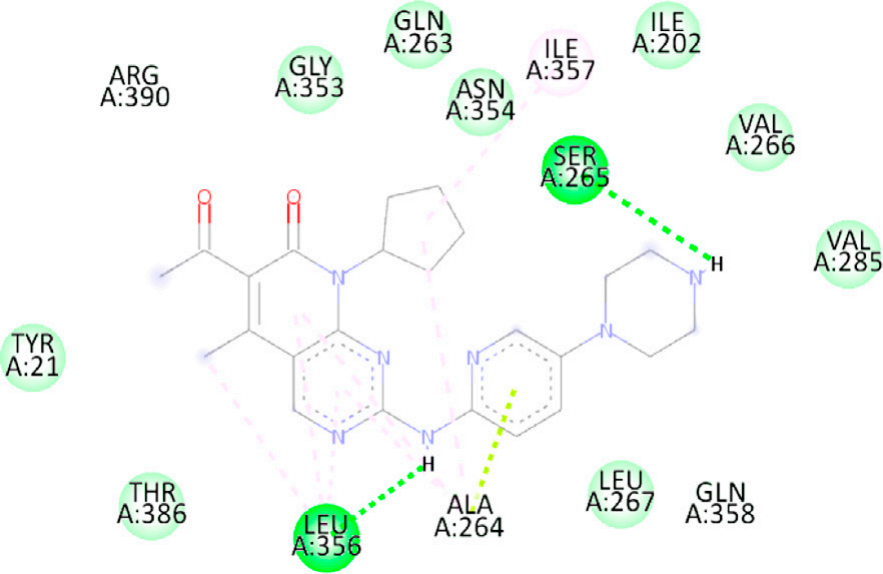
Pocket A			Ser265	H-bond	
			Leu356	H-bond	
Ribociclib	−7.07	6.65	Asp97	Sal bridge	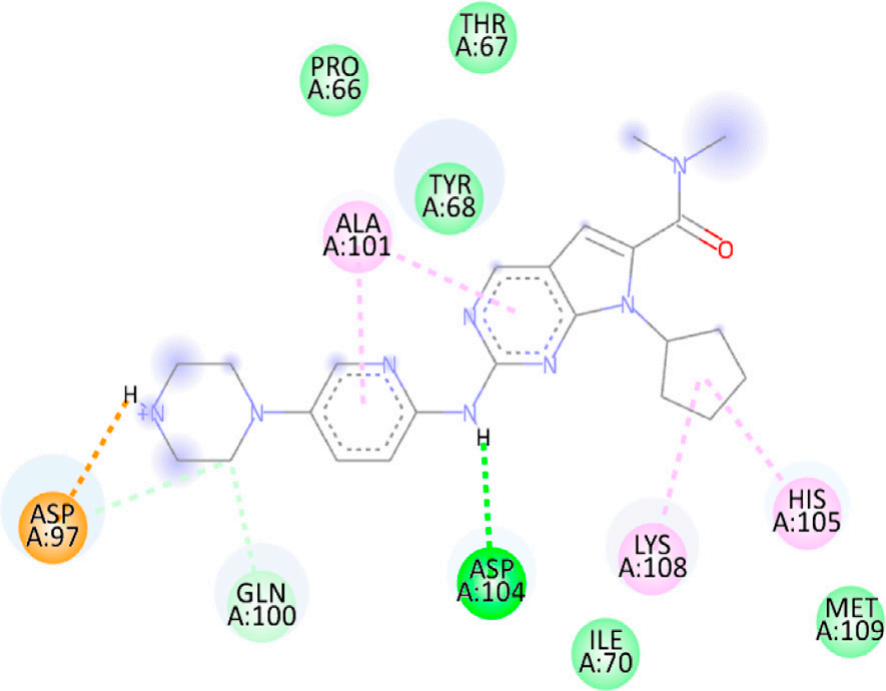
Pocket C			Asp104	H-bond	
Abemaciclib	−6.82	10.05	Lys62	2 Pi-cation	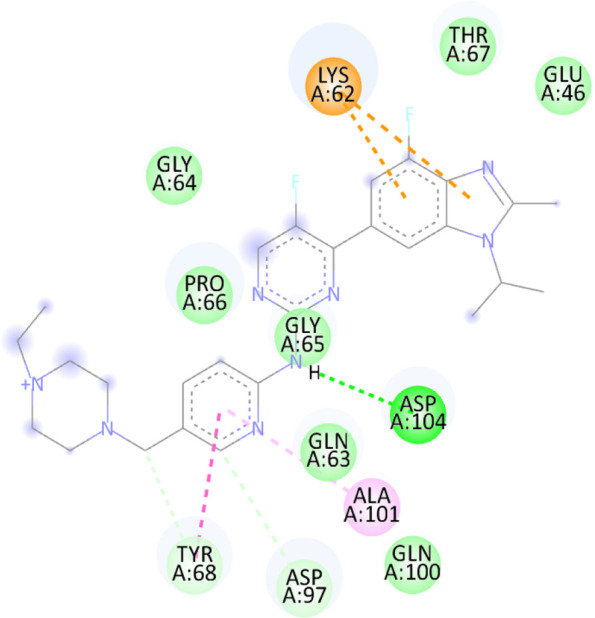
Pocket C			Tyr68	Pi-Pi	
			Asp 104	H-bond	

Romidepsin and Panobinostat preferred to bind to Pocket B with similar binding affinities: −6.52 and −6.49 kcal/mol, respectively. Romidepsin formed 3 H-bonds with Pocket B via 2 residues, Ser86 and Asn256, while more interactions were observed with Panobinostat mediated via 5 residues: Ser86, Asp95, Thr175, His176, and Asp200.

Finally, the last 4 small molecules could bind to Pocket C with binding energies (−7.07<∆G<−4.51 kcal/mol and inhibition constant 6.65<inhib. Const.<498.11 µM. Riboclib is reportedly the best hit towards Pocket C and the second-best hit against ACTL6A in general, followed by Abemaciclib.

### 3.11 Molecular dynamics simulations

**FIGURE 15:**
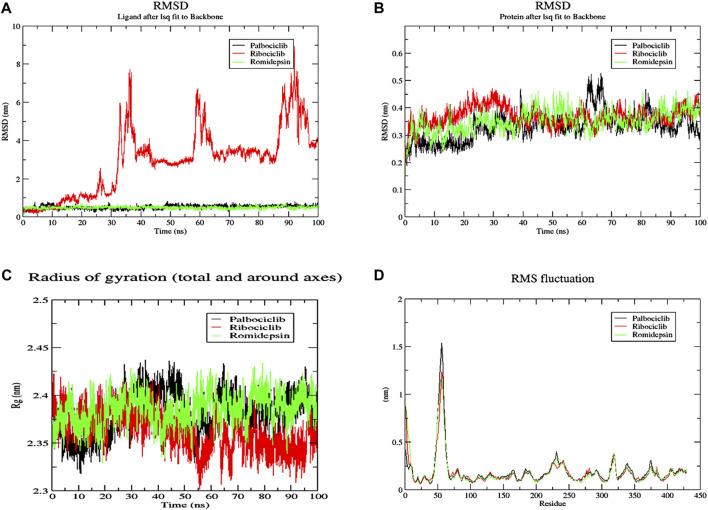
MD analysis of ACTL6A protein. **(A)**: The Root Mean Square Deviation (RMSD) for the protein **(B)**: RMSD for the ligands **(C)**: the Radius of Gyration (Rg) for the PTP1B catalytic pocket during a 100 ns MD simulation bound to the three active hits.

The conformational changes of protein-ligand complexes were examined using 2 methods: root-mean-square deviation (RMSD) and radius of gyration (Rg) analyses for both the ligand and target through the 100 ns MD simulation to assess the stability of the simulated system (Figure 15). These parameters were calculated after re-centering and re-wrapping the complex within the unit cells using the trjconv function of GROMACS. As shown in Figure 15A, the protein RMSD for the 3 complexes fluctuated between 0.25 and 0.45 nm after about 40 ns from the beginning of the simulation till its end. This low oscillation indicates the stability of the protein structure upon binding the 3 hits. The complexes’ radius of gyration (Rg), as a measure for its compactness, showed high compactness when the 3 hits bound to different pockets of ACTL6A (Figure 15C). The stability of this binding is studied by analyzing the RMSD of the 3 compounds while binding to the ACTL6A target (Figure 15B). Both structures, Palbociclib and Romidepsin, show very high stability, which was not the case with Ribociclib. A relatively stable oscillation is observed in the first 25 ns upon the binding of Ribociclib to Pocket B, followed by a high jump for about 10 ns (between 30 ns and 40 ns). This jump was observed again between 60 and 70 ns and between 85 and 95 ns After each of these jumps, a low fluctuation in Ribociclib RMSD is observed. Accordingly, further analyses were carried out to explain this pattern of oscillation.

**FIGURE 16:**
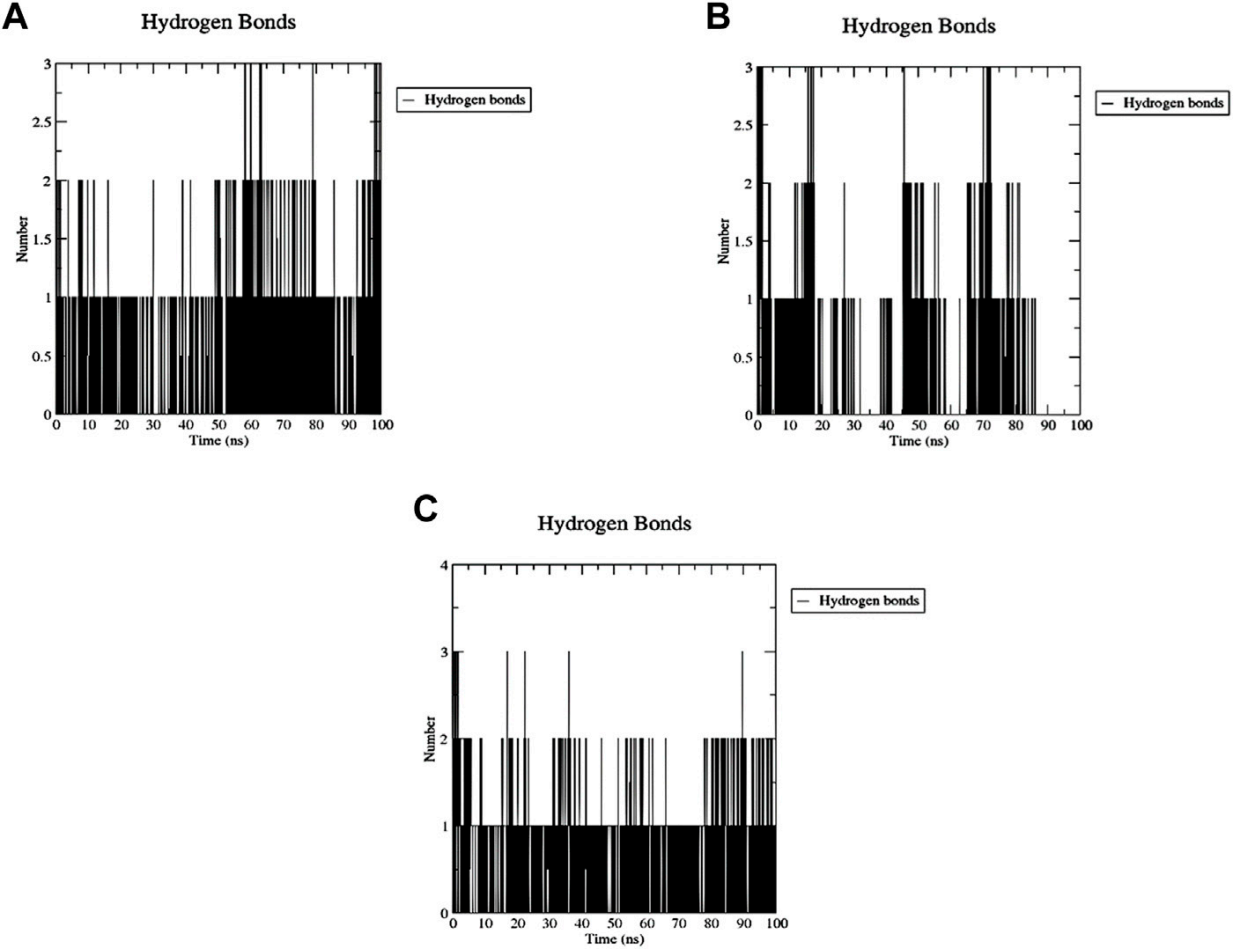
MD analysis of ACTL6A, highlighting the number of hydrogen bonds formed during the 100 ns MD trajectories with Palbociclib **(A)**, Ribociclib 12 **(B)**, and Romidepsin **(C)**.

The number of H-bonds formed during the 200 ns simulation was calculated to assess the stability of this binding (Figure 16). One stable H-bond is maintained during the whole trajectory for both Palbociclib and Romidepsin with the probability of forming 1-2 more H-bonds in some time points. On the other hand, Romidepsin failed to form stable H-bonds where no H-bonds were formed in periods 30–40 ns, 60–70 ns, and 85–100 ns. These time frames are the same ones where high ligand RMSD fluctuation was observed.

**FIGURE 17:**
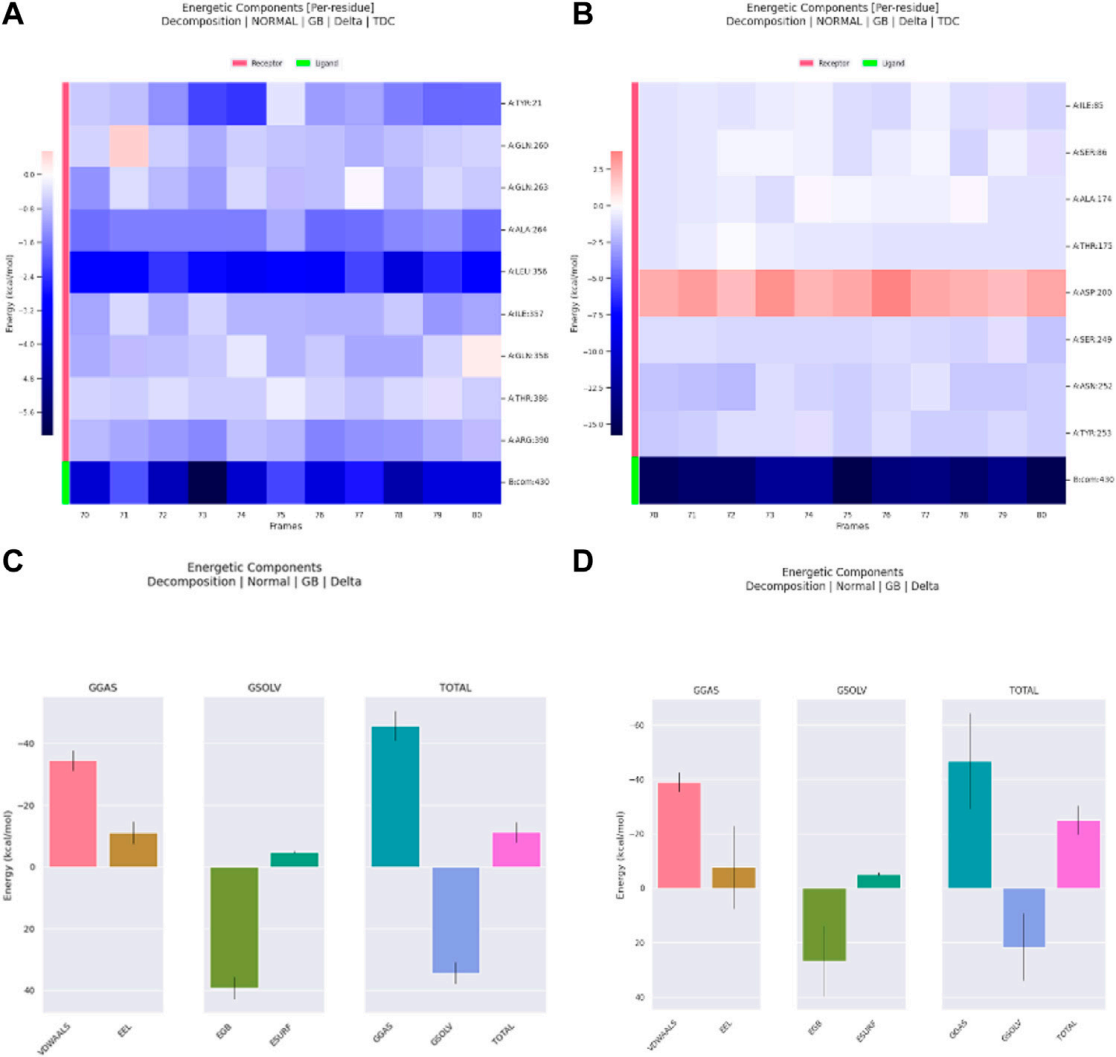
Residue-binding-free energy decomposition in 10 selected frames, Palbociclib **(A)**, Romidepsin **(B)**, and energetic component decomposition Palbociclib **(C)**, Romidepsin **(D)**.

The decomposition of energetic components per residue for the residues involved in the interaction with the hits was studied in selected time frames. It was studied for 10 stable frames between 70 and 80 ns for Palbociclib and Romidepsin. Similar residues to those reported in the molecular docking were remarked here. Leu356 and Ala264 show high binding energies maintained during the 10 selected frames. Similarly, Tyr21, Ile357, and Arg390 show good binding energies >−0.6 kcal/mol (Figure 17A). High contribution to hydrophobic interactions mediated via van der Waals is also noticeable with a total energy contribution of more than −30 kcal/mol (Figure 17C). Less energetic decomposition contribution is observed with Romidepsin (Figure 17D). Ser86, which is involved in the formation of 2 main H-bonds, shows a significant binding (around −2.5 kcal/mol) (Figure 17B). Like Palbociclib, high van der Waals energetic component (∼−40 kcal/mol) was reported which is suggested to be mediated by ILE85, Ala174, Thr175, Ser249, Asn252, and Thr253 due to their high energetic composition (Figures 17B, C).

**FIGURE 18:**
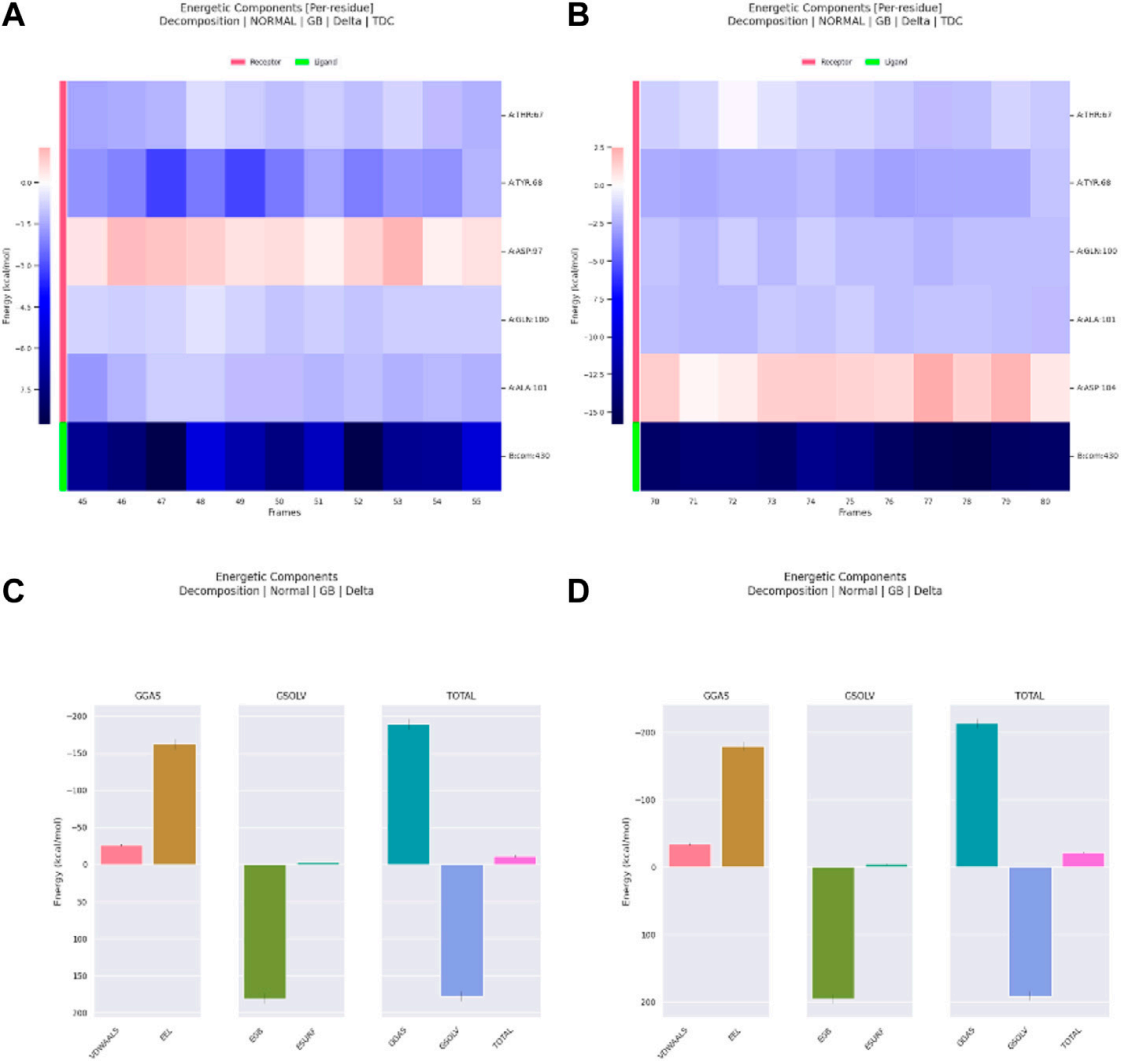
Residue-binding-free energy decomposition in 10 selected frames for Ribociclib, frames between 45 and 55 ns **(A)**, frames between 70 and 80 ns **(B)** and energetic component decomposition Ribociclib, frames between 45 and 55 ns **(C)**, frames between 70 and 80 ns **(D)**.

Meanwhile, for Ribociclib, frames between 45 and 55 ns and between 70 and 80 ns were selected for energetic components decomposition. Figures 18A, B show the residues responsible for binding Romidepsin and ACTL6A pocket C. In the first stable phase (after 45 ns), Figure 18A, the most significant residue is Asp97, which forms a salt-bridge with this hit structure, while when it was stabilized again after 70 ns (Figure 18B), this interaction was not observed and was replaced by the H-bond formed with Asp104. In both cases, the distribution of the energetic components is the same, where the electrostatic interaction surpasses the van der Waals energetic component (Figures 18C, D).

Though valuable, insights from MD simulations and MM/PBSA calculations are tempered by inherent limitations. The CHARMM36 force field introduces uncertainties, and a 100 ns duration may not fully cover conformational space—suggesting potential benefits from longer simulations. The interpretation of results in MM/PBSA necessitates caution due to its assumptions, which include neglecting solvent dynamics and overlooking entropy contributions. It is imperative to emphasize the importance of experimental validation in ensuring the reliability of findings. In conclusion, awareness of limitations, ongoing computational refinement, and complementary experimental data are essential for robust insights into Trichostatin A and Vorinostat binding dynamics with ACTL6A.

## 4 Discussion

ACTL6A, sometimes referred to as 53 kDa BRG-1/human BRM-associated factor (BAF53a), is crucially involved in multiple cellular processes such as vesicular transport, spindle orientation, nuclear migration, the cell cycle, and chromatin remodeling (Krasteva et al., 2012; Uhlen et al., 2015). Its involvement in tumorigenesis across multiple cancers has been widely reported (Meng et al., 2017). In particular, research conducted by Chen et al. (Chen et al., 2023) has established a correlation between ACTL6A expression and characteristics resembling those of cancer stem cells, highlighting its importance in the prognosis of ovarian cancer. Furthermore, ACTL6A has been associated with the inhibition of p21Cip1 promoter activity in epidermal squamous cell carcinoma, which contributes to the preservation of an aggressive phenotype (Shrestha et al., 2020). The complex interaction between ACTL6A and other crucial components has been clarified in HNSCC. In this context, ACTL6A and p63 collaborate effectively to control important genes, such as WWC1, which have an impact on oncogenic YAP activity and patient outcomes (Saladi et al., 2017). The significance of ACTL6A in ESCC is evident in its impact on cell cycle redistribution through the S6K1/pS6 pathway, which ultimately affects proliferation and DNA synthesis (Li et al., 2021). Furthermore, the role of ACTL6A in promoting the growth and spread of laryngeal squamous cell carcinoma has been associated with enhanced activation of YAP signaling (Dang et al., 2020). Molecular docking studies of ACTL6A have elucidated its interaction with essential biological pathways, offering significant insights that corroborate our findings with molecular modeling. These insights provide a deeper understanding of the structural and functional roles of ACTL6A in carcinogenesis. ACTL6A overexpression specifically enhances the migration and invasion of colon cancer cells, while reducing ACTL6A has the opposite impact under laboratory conditions (Zeng et al., 2018).

Gastric cancer (GC), renowned for its elevated probability of metastasis and mortality, demonstrates an intricate association with ACTL6A (Yang et al., 2023). Yang et al. (Yang et al., 2023) found that ACTL6A protects gastric cancer cells from ferroptosis by stimulating the production of glutathione. Nevertheless, the elevated amounts of ACTL6A in SCC cells resulted in almost complete occupancy of ACTL6A inside BAF complexes, hence intensifying the counteraction of polycomb proteins throughout the entire genome and triggering the activation of SCC genes (Chang et al., 2021). Suppressing the expression of ACTL6A in ovarian cancer cells in a laboratory setting led to a decrease in cell division, clonal expansion, and movement, indicating its potential as a target for therapeutic interventions (Zhang et al., 2019). This, in turn, contributes to the development of FSH-induced tumor formation (Zhang et al., 2019). Zhang et al. (Zhang et al., 2021) have shown that increased expression of ACTL6A is a prognostic risk factor in pancreatic cancer. Ji et al. (Ji et al., 2018) discovered that ACTL6A forms a physical association with YAP/TAZ and, in addition, interferes with the interaction between YAP and β-TrCP E3 ubiquitin ligase. This interference leads to the promotion of YAP protein breakdown. Additionally, Zhao et al. (Zhao et al., 2020) discovered that MiR-216a-3p inhibits the growth and spread of cervical cancer by reducing the activity of ACTL6A-mediated YAP signaling.

Several studies have attempted to examine the cancer-causing processes linked to ACTL6A in various types of tumors. However, there is a lack of extensive studies that thoroughly investigate the diverse effects of ACTL6A on many types of human tumors. The intricacies of the TME have been extensively described since it contains several elements that lead to the tumor’s development, the immune response to abnormal growth, the patient’s reaction to tumor management, and OS (Zabady et al., 2022). A comprehensive approach is necessary to establish a correlation between a single gene and the tumor’s progression, considering the problem’s complex nature. This necessitates analyzing the situation from multiple angles. Our in-depth molecular docking and structural analysis further support the significant role of ACTL6A as an oncogenic factor across multiple cancer types, suggesting potential targets for therapeutic intervention. The findings from molecular docking provided a robust framework for understanding the interaction dynamics of ACTL6A with potential inhibitors, which were analyzed *in silico*. This insight is crucial for future drug development strategies aimed at targeting ACTL6A in various cancers.

In order to achieve this goal, we utilized a comprehensive investigation across multiple types of cancer to examine the cancer-causing properties of ACTL6A. The study began by examining the ACTL6A distribution in different human tissues, which revealed its presence in multiple organs. A crucial characteristic of oncogenic proteins is their elevated expression in tumor tissue compared to normal tissue. Therefore, our following investigation focused on analyzing the differential expression of ACTL6A in various kinds of human malignancies. The results showed a significant upregulation of ACTL6A in the following tumor types: BLCA, BRCA, CESC, CHOL, COAD, ESCA, GBM, HNSC, LIHC, LUAD, LUSC, PRAD, READ, STAD, UCEC, DLBC, LGG, and THYM. Our research aimed to examine the potential correlation between the expression of ACTL6A and the stage and grade of cancer. The results of our study showed that LIHC, PAAD, and UCEC had an increase in both tumor stage and grade, which was associated with the expression of ACTL6A. Moreover, it has been noted that there is a direct correlation between the presence of ACTL6A and the spread of tumors in different organs, such as the breast, kidney, and liver. The previous differential comparison aimed to examine the amounts of ACTL6A protein in both normal and malignant tissues. Once again, it has been shown that there is a consistent pattern of elevated ACTL6A expression in tumor tissues across many types of malignancies, such as colon, clear cell RCC, HNSC, HCC, LUAD, LUSC, PAAD, GBM, and OV. The IHC staining results confirmed this discovery, demonstrating elevated levels of ACTL6A in the examined tumor tissues.

Survival analysis is crucial for studying disease progression and treatment efficacy (Nagy et al., 2021). We aim to determine the association between ACTL6A expression and OS in patients. The examination of the GEPIA database revealed a significant link between the ACTL6A expression and a less favorable prognosis in ACC, LGG, LIHC, and SARC. This was supported by the results of DFS and OS. KM plot analysis validated this positive connection across various cancer models, affirming ACTL6A’s potential as a prognostic biomarker in specified cancer types. Different genetic alterations are acknowledged as advantageous indications for the prognosis of human cancer. Prominent instances encompass the existence of mutant KRAS, which is linked to adverse results in pancreatic (Byers et al., 2012) and lung cancer (Shen et al., 2017), as well as the existence of mutated NRAS, which is connected with a bleak prognosis in metastatic melanoma (Jakob et al., 2012). Subsequent to this, our survival research investigated the impact of ACTL6A genetic alterations on patient survival, revealing a correlation between the presence of the ACTL6A genetic mutation and poor prognosis in both OS and DFS.

In the context of gene methylation status in human malignancies, prior research consistently highlights DNA hypermethylation deactivating tumor suppressor genes, while oncogenes undergo hypomethylation to promote malignancy. On the other hand, oncogenes frequently experience hypomethylation as a tactic to activate them, thereby facilitating the advancement of malignancies (Romero-Garcia et al., 2020). Significantly, the presence of hypomethylation in oncogenes such AQP1, LINE-1, and ELMO3 has been observed in salivary gland adenoid cystic carcinoma (Shao et al., 2011), colorectal cancer (Hur et al., 2014), and lung cancer (Soes et al., 2014), respectively. Consequently, a research project was initiated to examine the methylation patterns in the ACTL6A gene. The findings revealed decreased methylation levels in many types of tumors, specifically BRCA, HNSC, and READ, in comparison to their respective normal samples. In addition, the analysis of CpG aggregated methylation data emerged as a hindrance in CpG-aggregated methylation levels in BRCA, HNSC, THCA, and UCEC contrasted to their corresponding normal counterparts.

In recent decades, significant strides have been made in tumor immunotherapy, emerging as a widely acknowledged strategy in the fight against cancer (Peng et al., 2019). Immunotherapy drugs called ICIs, specifically αPD-1, have been authorized to treat various types of cancer in humans, including malignant melanoma, gastric carcinoma, and hepatocellular carcinoma (Chang et al., 2021). In order to further our comprehension within this particular framework, it was imperative to examine the correlation between heightened ACTL6A expression in tumor tissue and the existence of various immune cell types invading the tumor. Our investigation initially centered on the analysis of MDSCs, known to exert a favorable influence on the survival and dissemination of tumor cells (Condamine et al., 2015). Additionally, MDSCs stimulate angiogenesis in tumors and contribute to the formation of cancer stem cells (Weber et al., 2018). Consequently, the observed increase in MDSC infiltration demonstrated a connection with unfavorable clinical outcomes in cancer patients (Zhang et al., 2016). Furthermore, the control of DNA replication by ACTL6A may possibly have an interaction with immunological checkpoints in TME. Scientific studies have demonstrated that RFC4, a protein that plays a role in the processes of DNA replication and repair, is associated with immunological checkpoints and may be involved in the development of cancer (Alaa Eldeen et al., 2024). RFC4 is commonly shown to have an abundance of biological processes related to breast cancer, cell cycle, and DNA replication (Li et al., 2021). These findings indicate that ACTL6A-induced cancer development may influence immunological checkpoints by means of RFC4’s control over DNA replication and repair in the tumor microenvironment (TME). ACTL6A-induced carcinogenesis is characterized by the control of DNA replication and repair, which might potentially interact with immunological checkpoints in TME via RFC4’s regulation of DNA replication and repair. However, further research is required to completely understand the molecular pathways and processes by which ACTL6A-induced carcinogenesis interacts with immunological checkpoints in TME. Gaining a comprehensive understanding of these routes and processes is of utmost importance to design highly efficient cancer treatments.

However, additional inquiry is necessary to comprehensively examine the positive correlation between ACTL6A expression and invasion of MDSCs. The connection between ACTL6A overexpression and the NKT cell was investigated as the second cell type. This specific cell type plays a crucial role in Fighting early malignancies by participating in cancer immune surveillance and releasing many effector chemicals (Bae et al., 2019). The presence of NKT cells in tumor tissue has been found to be related to enhanced patient survival in several human malignancies, suggesting that these cells have tumor-suppressive properties (Wolf et al., 2018). An in-depth examination of the data revealed a positive correlation between ACTL6A and MDSC in BLCA, BRCA, CESC, COAD, ESCA, GBM, HNSC, KICH, KIRC, KIRP, LGG, LUAD, LUSC, PCPG, PRAD, SKCM, STAD, and THCA. In contrast, there was a negative connection detected between ACTL6A and NKT cells. By integrating the findings of ACTL6A expression with the infiltration of MDSC and NKT cells, it can be inferred that the heightened expression of ACTL6A may suggest an inadequate immune response to tumor proliferation. Furthermore, there is a direct relationship between LGG, KIRC, LIHC, and ACTL6A, as well as several immunological checkpoints. Furthermore, a positive correlation was observed between ACTL6A and various immune checkpoints in LGG, KIRC, and LIHC. Similarly, there was a positive correlation between ACTL6A and MSI in DLBC, KIRC, and SARC. Notably, a negative correlation was identified between ACTL6A and TBM within LUAD and SKCM. These findings underscore the complex interplay between ACTL6A expression and immune checkpoint regulation in specific cancer types, providing valuable insights into the potential implications for immunotherapeutic strategies.

Furthermore, our investigation of molecular interactions revealed that RUVBL1 was consistently found in both the “ACTL6A-interacting” and “ACTL6A-correlated” protein groups. Considering the well-established association of these proteins with a variety of human malignancies (Zhong et al., 2018), the mechanism by which they interact with ACTL6A presents itself as a very interesting target for the development of novel antitumor therapies. The outcomes of our investigation underscore the efficacy of machine learning models in utilizing ACTL6A-related protein expression profiles for LIHC detection. With high AUC values, ranging from 0.95 to 0.97 across LR, SVM, RF, and XGBoost, our models exhibited robust predictive capabilities. Notably, XGBoost demonstrated superior performance, achieving the highest AUC of 0.97. The ROC curve analysis further visually confirmed the models’ ability to discriminate between normal and primary tumor samples based on ACTL6A-associated protein expression. Our integrated approach, combining machine learning and enrichment analysis, provided insights into the molecular pathways associated with ACTL6A’s cancer-causing function.

In the quest for therapeutic interventions, we assessed the druggability of ACTL6A targets, revealing three potential druggable pockets (Pocket A, Pocket B, and Pocket C). The amino acid residues that make up the active site of these pockets are provided in Table S1 and depicted in Figure 14, colored in blue, yellow, and magenta, respectively. Each of our attached small molecules exhibited a preference for one of these three pockets in order to achieve the greatest possible fit. The observed binding interactions described in Table 1 indicate that all residues bound to Pocket A exhibited the ability to create 2 hydrogen bonds and a pi-lone pair interaction as shared characteristics. Palbociclib, Belinostat, and Roscovitine all fulfill the required interactions, although they have different binding energies: −8.09, −6.62, and −4.92 kcal/mol, respectively. The significant disparity suggests that hydrophobic interactions also have a crucial impact on enhancing the affinity of the hits toward their pocket. However, no shared key interactions were seen when binding with Pocket B, except for the involvement of Ser86. Romidepsin and Panobinostat had similar binding energies, with values of −6.52 and −6.49 kcal/mol, respectively. Additionally, they both had similar projected inhibition constants, with values of 16.49 and 17.56 µM, respectively.

Pan-CDK inhibitors exhibit superior binding affinity towards Pocket C, particularly Ribociclib, Abemaciclib, and flavopiridol, demonstrating excellent binding affinities. In contrast, Vorinostat (a histone deacetylase inhibitor) has weaker binding energy and little binding interactions. The residues Lys62 and Tyr68 are frequently found in 3 of the 4 hits bound to Pocket C. This binding occurs through the formation of Pi-cation, Pi-Pi, and H-bond interactions. Upon the binding of both Ribociclib and Abemaciclib, the creation of a hydrogen bond with Asp104 was seen. These two compounds were shown to be the most effective. This indicates that the interaction with Asp104 enhances the binding affinity of the hits. A further significant interaction has been reported: the formation of a salt bridge between Ribociclib and Asp97. This interaction is believed to be responsible for the strong binding affinity and inhibition of Ribociclib against ACTL6A.

Palbociclib, Ribociclib, and Romidepsin exhibited the highest docking binding affinities towards the ACTL6A target, demonstrating their capability to attach to three distinct anticipated pockets: A, C, and B, respectively. Palbociclib and Romidepsin exhibited exceptional stability throughout a 100-nanosecond molecular dynamics trajectory investigation. Both exhibit a high degree of backbone compactness, accompanied by minimal variability in RMSD. Palbociclib could retain at least one hydrogen bond from the interactions identified in the molecular docking analysis. The investigation of energy decomposition of residues showed that the stable contact is mostly contributed by the hydrogen bond created by Leu356, followed by other hydrophobic interactions such as the Pi-lone pair interaction with Ala264. Furthermore, Van der Waals interactions provide three times as many benefits as electrostatic interactions. Romidepsin maintained its strong binding affinity by forming hydrogen bonds with Ser86 and engaging in numerous van der Waals interactions with the hydrophobic residues in the pocket.

Our study concludes that ACTL6A has a diverse impact on several types of human tumors, providing a thorough grasp of its effects. The combination of molecular, clinical, and therapeutic factors highlights ACTL6A’s importance as a possible predictive biomarker and therapeutic target in different malignancies. The molecular connections that have been uncovered and the druggable pockets that have been found offer exciting opportunities for future study and the creation of novel anti-cancer therapies. Future research should prioritize the analysis of the intricate mechanisms that underlie the involvement of ACTL6A in the development of tumors, as well as the exploration of its potential as a target for precise medical interventions in cancer treatment.

## 5 Limitations and future directions

In concluding our study, it is essential to recognize and address several inherent limitations that may impact the interpretation and generalizability of our findings. Firstly, the reliance on bioinformatics analyses and publicly available databases introduces potential biases and emphasizes the need for cautious interpretation, highlighting the necessity of future experimental validations. Additionally, the observed correlations between ACTL6A expression and clinical outcomes should be interpreted as associations rather than implying causation, underscoring the imperative for mechanistic studies to elucidate the functional consequences of ACTL6A dysregulation in tumorigenesis. While the pan-cancer analysis provides a comprehensive overview, its limitations in capturing cancer type-specific nuances warrant future investigations focusing on individual cancer types. Furthermore, our study predominantly focused on ACTL6A overexpression, necessitating experimental validations to establish the functional consequences in cancer cells. Lastly, the chemoinformatics approach for identifying potential ACTL6A inhibitors represents a preliminary step, emphasizing the need for extensive experimental validations, including *in vitro* assays and preclinical studies, to assess the therapeutic potential of the identified candidates accurately. Acknowledging these limitations enhances the transparency of our findings and provides valuable insights for guiding future research endeavors.

## 6 Conclusion

This study thoroughly examined multiple biological data sets to investigate the role of ACTL6A in tumor growth. Here, we have discovered that ACTL6A is consistently expressed at higher levels in tumor tissues compared to normal tissues. Notably, this excessive expression showed a connection with an advanced stage and grade of malignancies, as well as unfavorable clinical results in different types of human tumors. Furthermore, it was shown that genetic modifications in ACTL6A can be used to forecast a decline in patient survival. The function of ACTL6A encompasses the regulation of immune cell infiltration, namely facilitating the infiltration of immunosuppressive cells in the TME. ACTL6A, due to its oncogenic characteristics, is a promising target for antitumor therapy. In our investigation, we also used a chemoinformatics technique to assess several inhibitors of ACTL6A. This allowed us to discover potential compounds promising to intervene in tumor growth. These first results establish a basis for future wet lab investigations to confirm and investigate the therapeutic possibilities of targeting ACTL6A.

## Data Availability

The raw data supporting the conclusion of this article will be made available by the authors, without undue reservation.
